# Tissue-wide metabolomics reveals wide impact of gut microbiota on mice metabolite composition

**DOI:** 10.1038/s41598-022-19327-w

**Published:** 2022-09-02

**Authors:** Iman Zarei, Ville M. Koistinen, Marietta Kokla, Anton Klåvus, Ambrin Farizah Babu, Marko Lehtonen, Seppo Auriola, Kati Hanhineva

**Affiliations:** 1 Institute of Public Health and Clinical Nutrition, School of Medicine, Faculty of Health Science, University of Eastern Finland, P.O. Box 1627, 70211 Kuopio, Finland; 2 Food Chemistry and Food Development Unit, Department of Biochemistry, University of Turku, Itäinen Pitkäkatu 4, 20014 Turku, Finland; 3 School of Pharmacy, Faculty of Health Science, University of Eastern Finland, P.O. Box 1627, 70211 Kuopio, Finland; 4 LC-MS Metabolomics Center, Biocenter Kuopio, 70211 Kuopio, Finland

**Keywords:** Biochemistry, Cell biology, Molecular biology, Biomarkers, Diseases

## Abstract

The essential role of gut microbiota in health and disease is well recognized, but the biochemical details that underlie the beneficial impact remain largely undefined. To maintain its stability, microbiota participates in an interactive host-microbiota metabolic signaling, impacting metabolic phenotypes of the host. Dysbiosis of microbiota results in alteration of certain microbial and host metabolites. Identifying these markers could enhance early detection of certain diseases. We report LC–MS based non-targeted metabolic profiling that demonstrates a large effect of gut microbiota on mammalian tissue metabolites. It was hypothesized that gut microbiota influences the overall biochemistry of host metabolome and this effect is tissue-specific. Thirteen different tissues from germ-free (GF) and conventionally-raised (MPF) C57BL/6NTac mice were selected and their metabolic differences were analyzed. Our study demonstrated a large effect of microbiota on mammalian biochemistry at different tissues and resulted in statistically-significant modulation of metabolites from multiple metabolic pathways (p ≤ 0.05). Hundreds of molecular features were detected exclusively in one mouse group, with the majority of these being unique to specific tissue. A vast metabolic response of host to metabolites generated by the microbiota was observed, suggesting gut microbiota has a direct impact on host metabolism.

## Introduction

Microbiota (microbial community) or microbiome (collective genome of microbial community) is defined as the commensal, symbiotic, and pathogenic microbial community including bacteria, fungi, archaea, algae, and small protists which reside inside and on the host body^1–3^. Some of our tissues, such as those with a mucosal membrane, contain highly adapted and evolved microbial consortia^4^, with the vast majority of the microbiota within our gastrointestinal (GI) tract because of its nutrient-rich environment. Gut microbiota has a complex influence on human physiology and nutritional status^5,6^ by influencing the absorption, metabolism, and storage of ingested nutrients and by producing a diverse array of metabolites. Examples include digestion and bioconversion of food components such as hydrolysis and fermentation of indigestible plant nutrients (e.g., oligo- and polysaccharides known as microbiota-accessible carbohydrate or in short MAC) to make them bioavailable to the host^7–9^ and subsequently, production of metabolites involved in energy homeostasis, namely short-chain fatty acids (SCFAs)^10,11^; biosynthesis of indoles, aromatic amino acid metabolites, vitamins, and sphingolipids^12–14^; cholesterol synthesis inhibition and bile acid biotransformation by regulating their composition, abundance, and signaling^15–17^; stimulation and regulation of the immune system as well as inhibition of pathogens (e.g., production of antimicrobial compounds, regulating of intestinal pH, and competition for ecological niche) which in the end leads to support of intestinal function^18–20^; removal of toxins, drug residues and carcinogens from the body^21,22^; and even potential regulation of host central nervous system^23–25^.

Despite the fact that the mature microbiota is very resilient, high inter-individual variability in the composition of human gut microbiota can be explained by internal and external stimulants such as age, genotype, mode of delivery, antibiotic use, diet, demography, lifestyle, social interactions, stress, and environmental exposure to various xenobiotics^26–28^. Diet alone is one of the most important modifiable lifestyle factors contributing to variation in gut microbiota composition, and for instance, the impact of diet on gut microbiota is found to be higher as compared to genotype^29^. Furthermore, the inter-individual variation might explain why the impact of nutritional interventions varies among individuals, even though the same food was consumed^30^. Given that the differences in commensal microbiota and even reduced microbial diversity may impact human health and disease, changes in the composition of gut microbiota are linked to the development of many disorders such as type 2 diabetes, cardiovascular dyslipidemia, and cirrhosis, cancer, allergies, inflammatory bowel disease (IBD), neurodevelopmental disorders (e.g., autism), aging, and many more^31–33^. Therefore, manipulation of gut microbiota, with the aim of preventing and treating chronic diseases, can lead us to a deeper systematic understanding of the microbiota-host axis. Subsequently, this will enable us to bridge the gap toward tailored dietary approaches for personalized treatment, by incorporating nutrient-microbiota-host interactions^34^.

Metabolomics is a well-established and powerful tool that can be applied to identify microbiome-derived or microbiome-modified metabolites and to better understand the modulation of microbiota and how it affects the metabolism in a host^35–39^. Metabolomics can also help to define the metabolic interactions among the host, diet, and gut microbiota^40^. When applied to various tissues from murine models, it is shown that numerous metabolite classes have been affected by the germ-free status, especially energy metabolism, lipids (e.g., fatty acids, phosphatidylcholine, and carnitines), oligopeptides, followed by amino acid metabolism and its derivatives (e.g., glutamates, indoles, lysine, aromatic, polyamines), and nucleoside^37,39^. Nonetheless, our knowledge of microbial modulation of host overall metabolism is limited. In particular, it is not clear how the gut microbiota affects systemically, the metabolically-important organs. Given the complexity of systemic host overall metabolism, it is clear that a multi-tissue approach is needed to clarify these issues. We herein hypothesized that gut microbiota influences the overall biochemistry of the host metabolome and its effect is tissue-specific. Thus, the aim of the study was to establish a comparative metabolite-level overview of 13 different tissues from germ-free (GF) and conventionally-raised mice (murine-pathogen-free, MPF) affected by the intestinal microbial community using non-targeted metabolite profiling approach. We chose to analyze multiple tissues because it provides an excellent opportunity to assess the extent of the interplay between bacterial metabolic and systemic human pathways. The metabolite composition of plasma, heart, liver, pancreas, muscle, duodenum, jejunum, ileum, cecum, colon, visceral adipose tissue (VAT), subcutaneous adipose tissue (SAT), and brown adipose tissue (BAT) were analyzed, and demonstrated a massive metabolic impact across all the tissues studied. We observed significantly large number of chemical species in the tissues because of the presence of the microbiota, and a range of 29–74% of all detectable metabolites varied in abundance by at least 50% between the 2 mouse lines. Our study confirms earlier findings related to specific metabolites known to be of microbial origin, such as indolepropionic acid (IPA), and trimethylamine N-oxide (TMAO), as they were absent from the GF mice tissue. Interestingly, our study demonstrated the organ-wide distribution of these metabolites. For example, IPA was detected in plasma, colon, and cecum and TMAO was detected in all the examined tissues except liver and duodenum (data for TMAO is not shown in this manuscript)﻿. We also observed the lowest number of detected metabolites in plasma, among all the 13 tissues, which highlights the importance of the metabolically-active organs. Despite this fact, plasma was amongst the most affected tissues in the GF mice, and it showed higher percentage of significantly abundant metabolites in this mouse group when compared to their counterpart plasma in the MPF mice. Furthermore, as novel finding, we showed large scale differences in small peptide metabolism, fatty amides, and polyamines across multiple tissues in germ-free mice compared to the conventionally-raised mice that to our knowledge, no other studies have addressed yet. In a broader perspective, we also observed that the absence of a gut microbiota is reflected by increased levels of arginine and proline metabolism (urea cycle), oligopeptides, carbohydrates and energy metabolism, and secondary metabolites (flavonoids, phenolic acids, and terpenes), and decreased levels of bile acids, acylcarnitines, fatty amides, aromatic amino acids, lysine and polyamine metabolisms.

## Results

### The impact of microbiota on the metabolome of GF and MPF mice was tissue-wide

**Figure 1:**
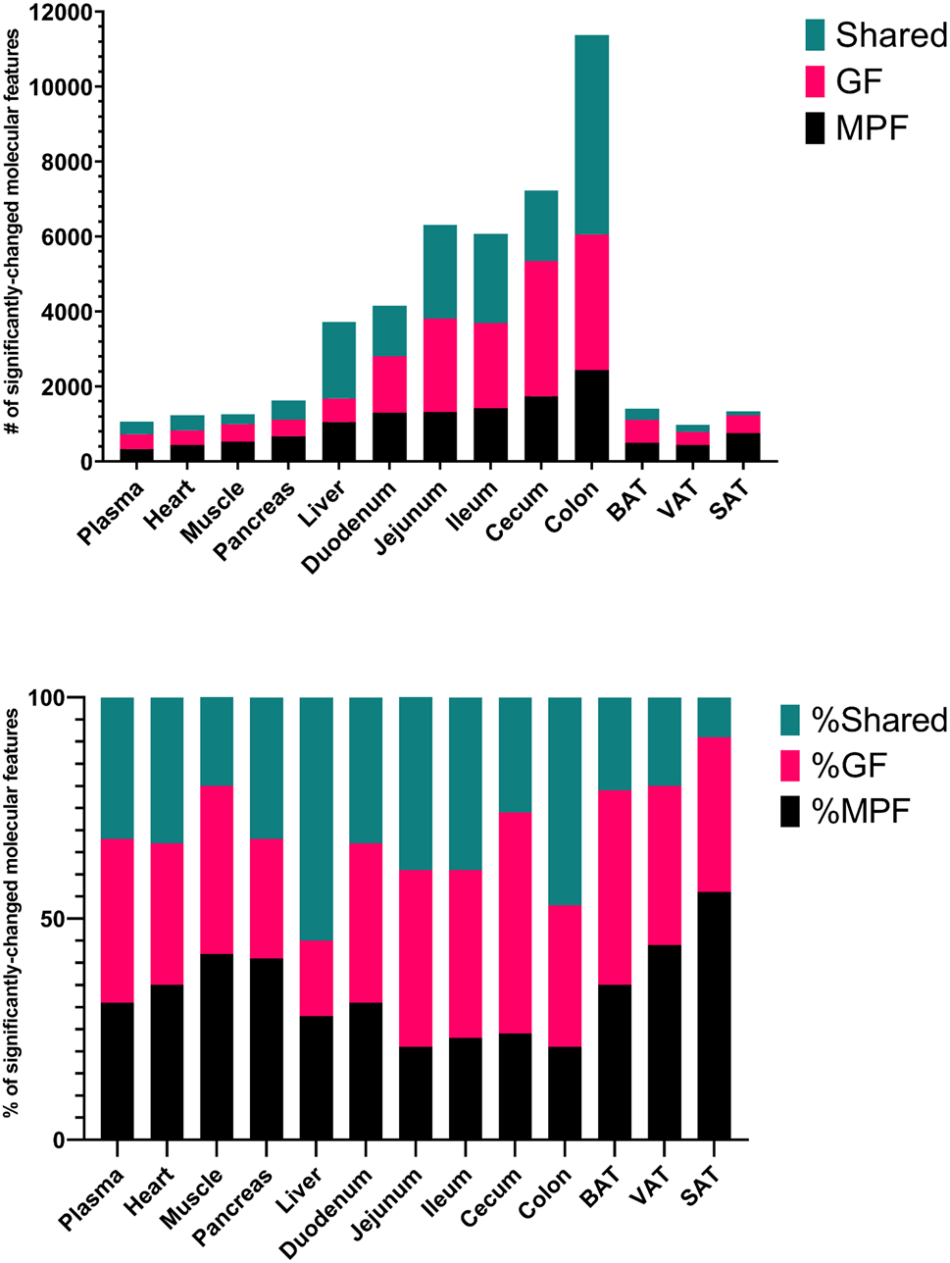
Number and percentage of significant molecular features per tissue. The total number of total significant molecular features from each tissue sourced from MPF only, GF only or shared (upper), Percentage of significant unique molecular features from each murine class per tissue (lower). Level of significance is defined as having a fold change ≥ 1.3, *p* value ≤ 0.05, and *q* value ≤ 0.05.

A total of 130 samples from 13 tissues were analyzed from five GF and five MPF mice using high-resolution LC–MS platform with four analytical modes employing RP and HILIC modes with both positive and negative ionization providing an initial wide-scale assessment of the effect of the microbiome on mammalian metabolism. Figure 1 and Supplementary Table 1 summarize the detection rate and the proportion of differential and unique molecular features in the 13 different tissues; most of detected individual features were present in the GI tract tissues (i.e., duodenum, jejunum, ileum, cecum, and colon), and the liver (≃ 41 to 74%), with the cecum containing highest percentage of all detected features, whereas plasma with 23% of all detected features showed the lowest number among all the 13 tissues. The GI tract tissues, and the liver also showed the highest percentage of significant molecular features (≃ 26 to 64%) (according to the statistical criteria applied) with the cecum containing highest percentage of significant molecular feature. It is noteworthy that the GF mice showed the highest percentage of significant molecular features (≥ 50%) in all the tissues from the GI as well as plasma and BAT when compared to the MPF mice in the same tissue. Contrarily, MPF mice showed the highest percentage of significant molecular features in the liver, SAT, pancreas, muscle, VAT, and heart. Colon and SAT were the only tissues with more than half of their detected molecular features unique to the GF and MPF, respectively.

**Figure 2:**
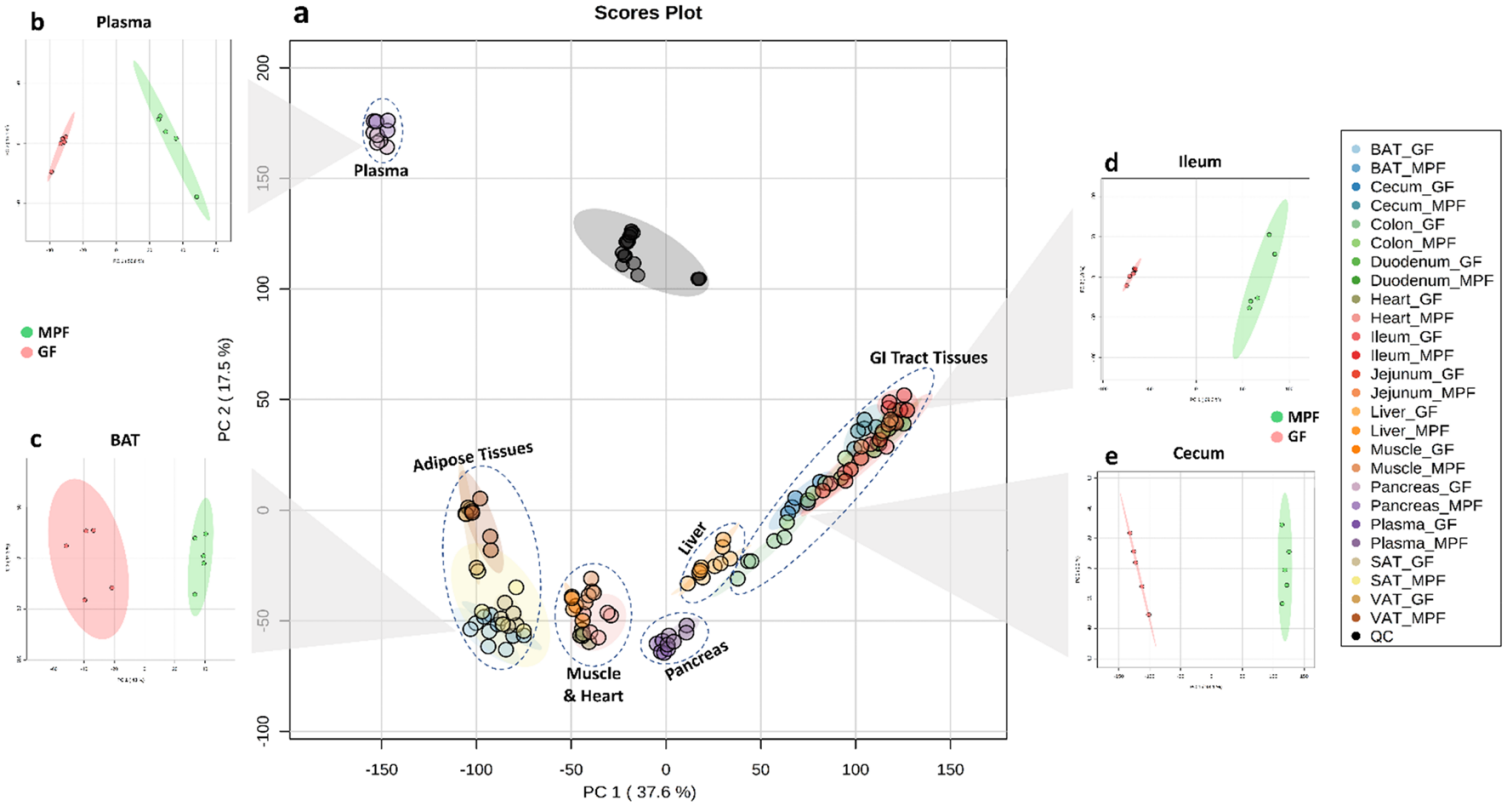
Principal component analysis (PCA) of the profiling data shows separation between tissues and mice groups. Data shown for reverse phase (RP) and HILIC modes with both positive and negative ionization; (**a**) PCA of all the analyzed samples from all tissues, (**b**) PCA of plasma samples from the GF and MPF mice, (**c**) PCA of BAT samples from the GF and MPF mice, (**d**) PCA of ileal samples from the GF and MPF mice, (**e**) PCA of cecal samples from the GF and MPF mice.

To assess the differences in the metabolite composition in the GF and MPF mice, we performed a principal component analysis (PCA) on the molecular features across all the tissues. The metabolic profile was influenced by the tissue type as the main driving factor as well as by the colonization status (i.e., GF or MPF), indicating that the extensive effect of microbiota on the metabolite composition extends to peripheral organs (Fig. 2).

**Figure 3:**
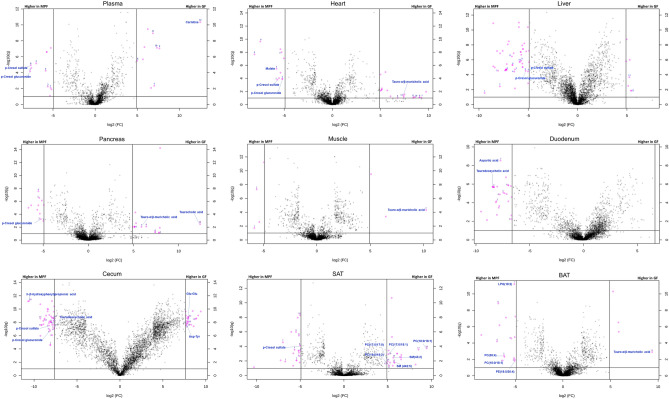
Volcano plots of the molecular features detected in nine representative tissues. The illustrated tissues include plasma, heart, liver, pancreas, muscle, duodenum, cecum, subcutaneous adipose tissue (SAT), and brown adipose tissue (BAT); see Supplementary Fig. 1 for volcano plots of all studied tissues individually with selected metabolites annotated. The binary logarithm of the fold change (FC) is shown as the function of the negative common logarithm of the q value (false discovery rate corrected p value). A positive log2(FC) signifies a higher abundance in the GF mice compared to the MPF mice. The purple dots represent molecular features fulfilling the significance criteria (FC > 200 or FC < 0.005 for the cecum and the colon tissues, FC > 100 or FC < 0.01 for duodenum, jejunum, and ileum, FC > 30 or FC < 0.033 for the rest of the tissue types, and q < 0.1). Molecular features are presented as their binary logarithmic fold change [log2(FC)]against the negative common logarithm of the q value [false discovery rate corrected p value; − log10(q)] of the differential expression between the GF and MPF mouse group. Although the purple dots represent molecular features fulfilling the above-mentioned significance criteria, and were unique to the sample type.

Further detailed investigation on the differential molecular features in each tissue, demonstrates how the magnitude of the impact of the colonization status varies across the different tissue types (Fig. 3, Supplementary Fig. 1). Likewise, on the level of individual metabolites and metabolite classes, it was evident that the variation across organs was high, as some of metabolites were differential only in one tissue and some others across several, for example tauro-alpha/beta-muricholic acid and *p*-cresol glucuronide were differential in multiple tissues and present only in GF and MPF mice respectively, whereas particular phospholipids were only differential in SAT and mostly present in GF mice (Fig. 3; Supplementary Fig. 1).

**Figure 4:**
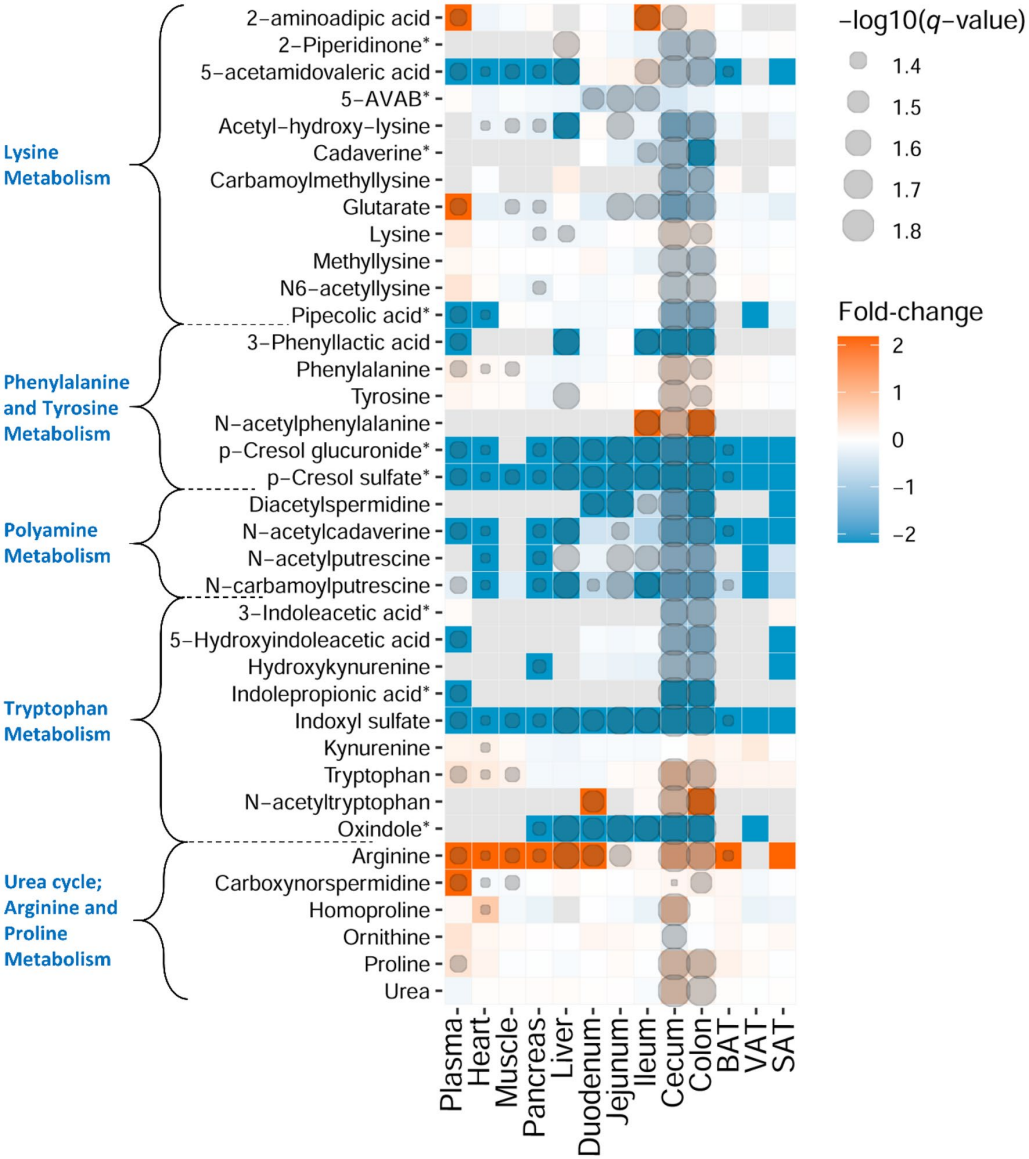
Heatmap representation of identified metabolites in amino acid chemical class. Fold-change (GF vs. MPF) and degree of significance comparisons were performed between the GF and MPF within each tissue (Mann–Whitney U-test and Benjamini and Hochberg false discovery rate correction *p* value ≤ 0.05, and *q* value ≤ 0.05). Each comparison for a tissue is represented by a colored cell. Gray cells represent metabolites that were not found in the tissue. Orange and blue cells represent metabolites more abundant in GF and MPF mice, respectively. *Metabolite is known to be bacterial-borne.

**Figure 5:**
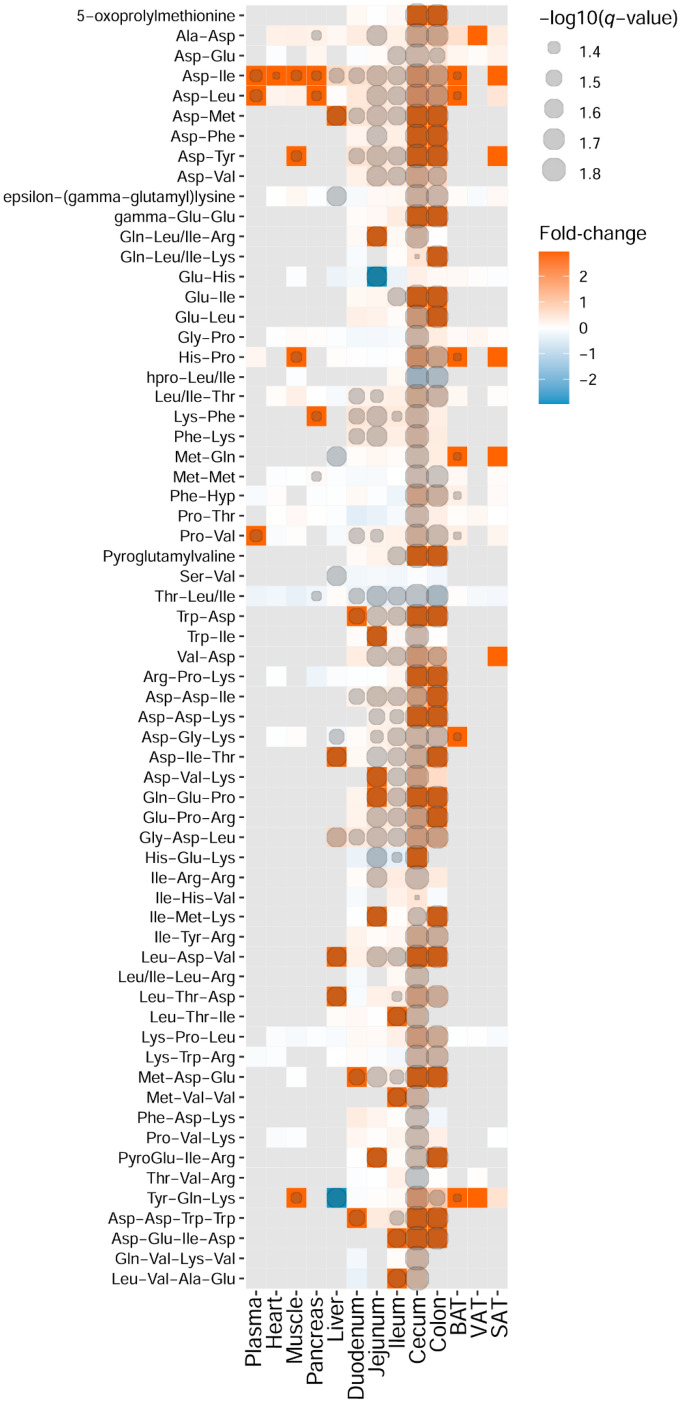
Heatmap representation of identified metabolites in peptide chemical class. Fold-change (GF vs. MPF) and degree of significance comparisons were performed between the GF and MPF within each tissue (Mann–Whitney U-test and Benjamini and Hochberg false discovery rate correction *p* value ≤ 0.05, and *q* value ≤ 0.05). Each comparison for a tissue is represented by a colored cell. Gray cells represent metabolites that were not found in the tissue. Orange and blue cells represent metabolites more abundant in GF and MPF mice, respectively.

**Figure 6:**
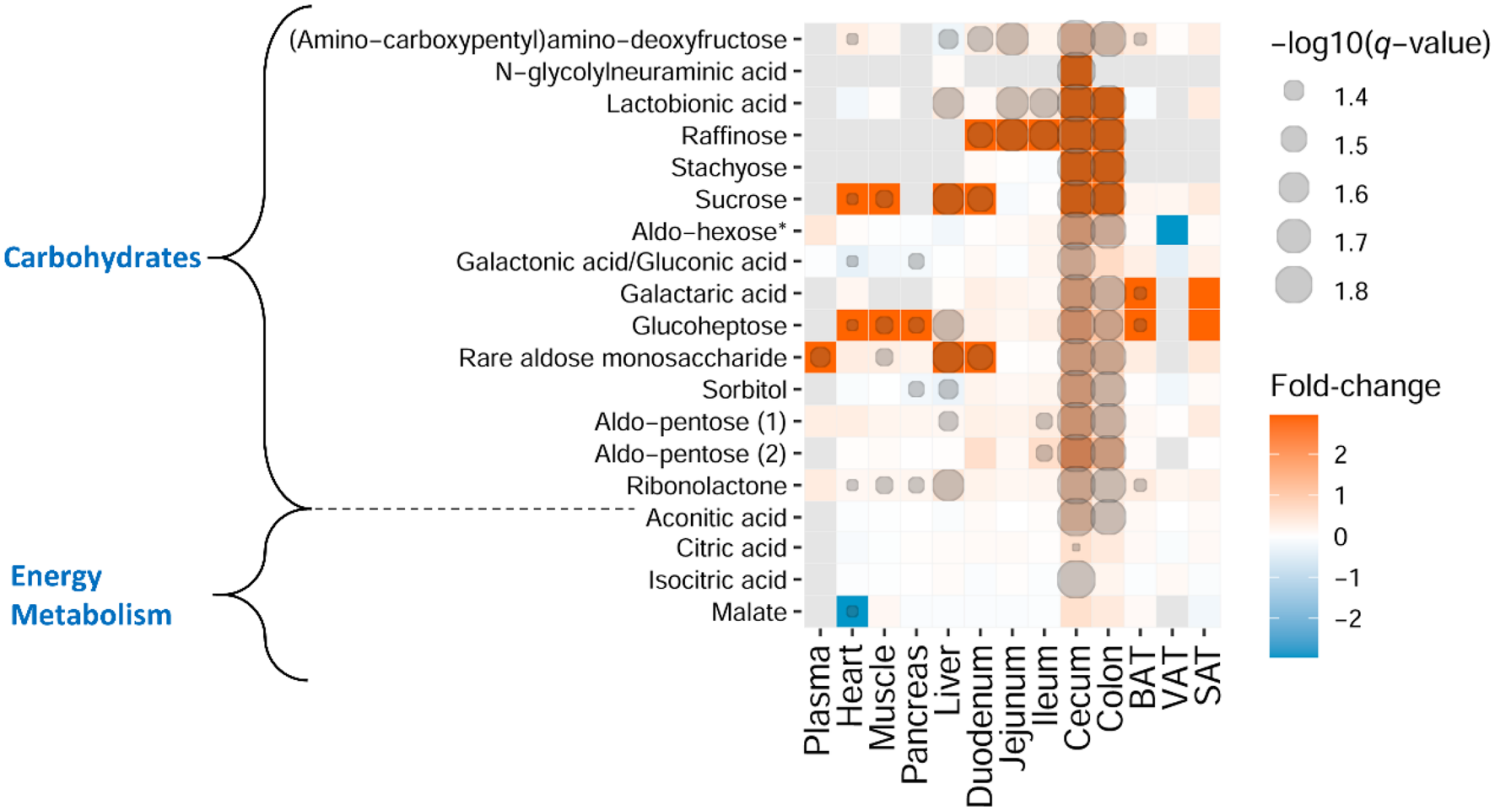
Heatmap representation of identified metabolites involved in carbohydrate and energy metabolism. Fold-change (GF vs. MPF) and degree of significance comparisons were performed between the GF and MPF within each tissue (Mann–Whitney U-test and Benjamini and Hochberg false discovery rate correction *p* value ≤ 0.05, and *q* value ≤ 0.05). Each comparison for a tissue is represented by a colored cell. Gray cells represent metabolites that were not found in the tissue. Orange and blue cells represent metabolites more abundant in GF and MPF mice, respectively. *This metabolite is one the following isomers: Mannitol, Galactitol, Iditol.

**Figure 7:**
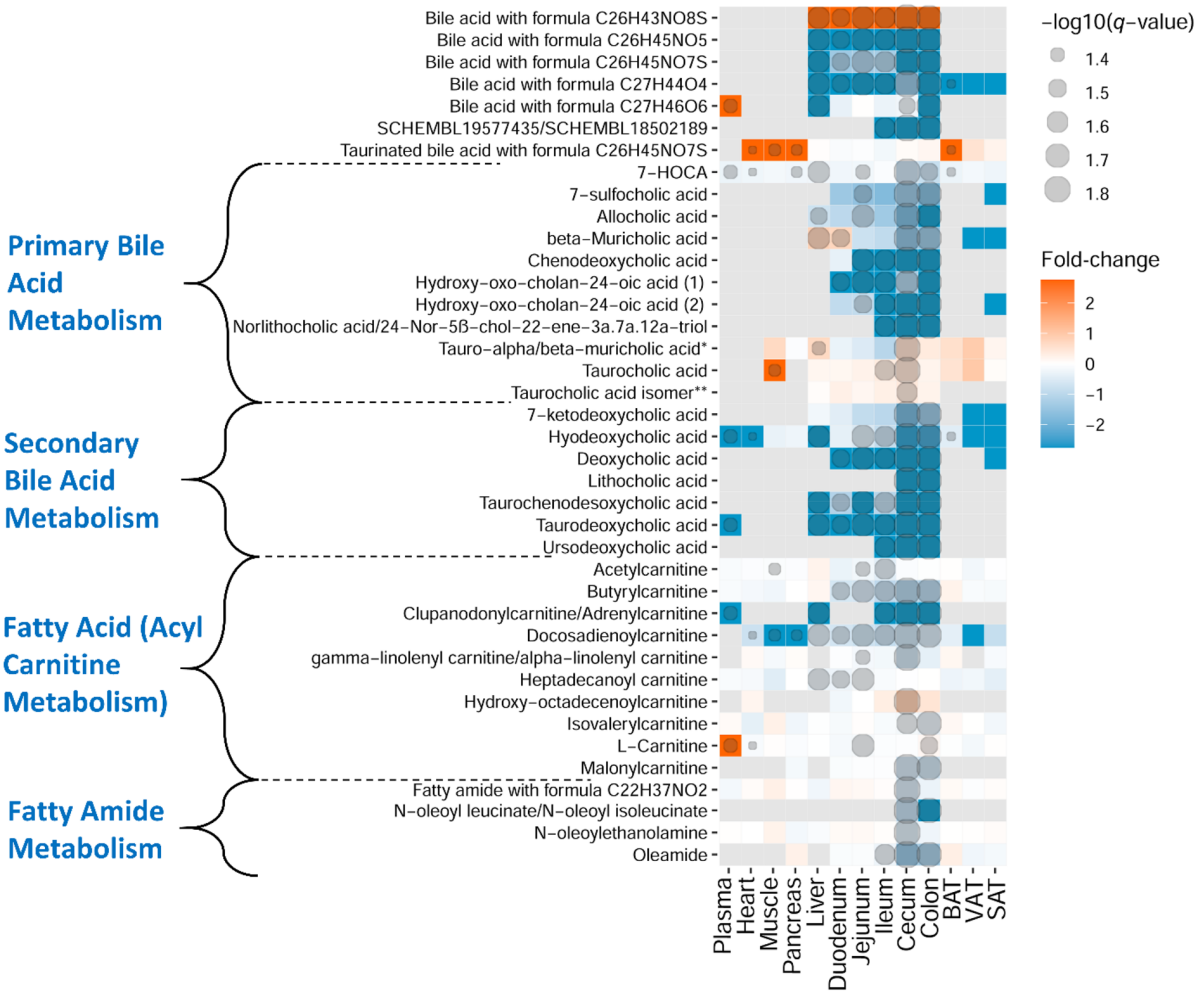
Heatmap representation of identified metabolites in bile acids, fatty amides, carnitine, and acylcarnitine metabolism classes. Fold-change (GF vs. MPF) and degree of significance comparisons were performed between the GF and MPF within each tissue (Mann–Whitney U-test and Benjamini and Hochberg false discovery rate correction *p* value ≤ 0.05, and *q* value ≤ 0.05). Each comparison for a tissue is represented by a colored cell. Gray cells represent metabolites that were not found in the tissue. Orange and blue cells represent metabolites more abundant in GF and MPF mice, respectively. *Despite comparing the spectra against the purified standard, we were not able to differentiate between tauro-α-muricholic acid and tauro-β-muricholic acid. **Taurocholic acid isomer: either taurallocholate or tauroursocholate or taurohyocholate.

**Figure 8:**
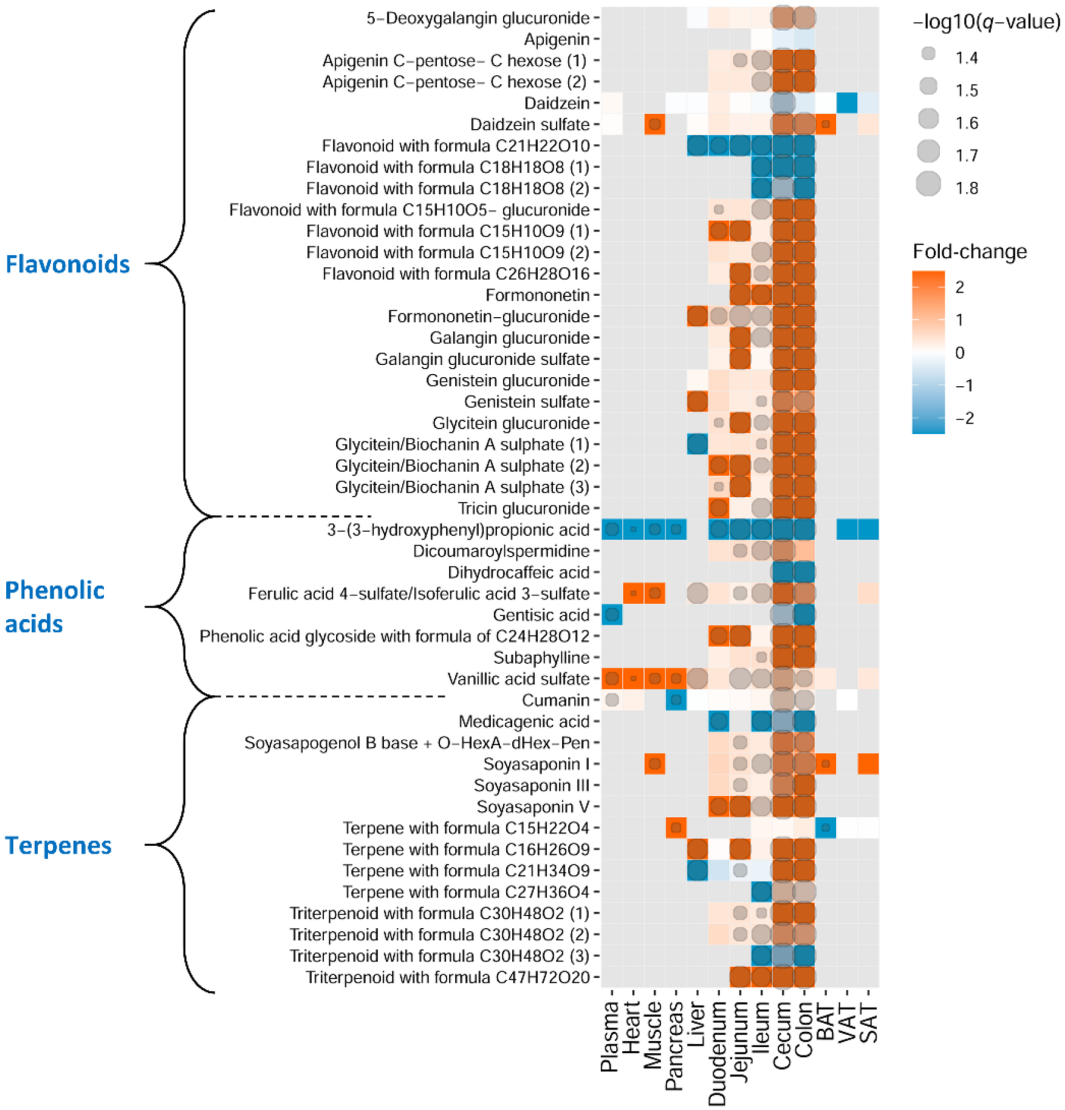
Heatmap representation of identified metabolites in phenolic acid derivatives, flavonoids, and terpenes. Fold-change (GF vs. MPF) and degree of significance comparisons were performed between the GF and MPF within each tissue (Mann–Whitney U-test and Benjamini and Hochberg false discovery rate correction *p* value ≤ 0.05, and *q* value ≤ 0.05). Each comparison for a tissue is represented by a colored cell. Gray cells represent metabolites that were not found in the tissue. Orange and blue cells represent metabolites more abundant in GF and MPF mice, respectively.

We further applied *k*-means cluster analysis to examine the abundance of the differential molecular features between the mice groups across all tissue types. In our data, the molecular features were clustered based on (1) their presence in the different tissues, (2) their presence in the GF or MPF mice (Supplementary Fig. 2). Among the 10 clusters generated by *k*-means clustering analysis, clusters 2, 4, and 5 (1214 molecular features in total) contained the metabolites that showed significant differences between the GF and MPF mice that were unique to one tissue or a subset of tissues. Cluster 2 indicates the subset of molecular features that were significantly higher in the cecum and the colon of the GF mice. Cluster 4 represents a subset of molecular features that were significantly higher in the cecum and the colon of the MPF mice. Cluster 5 shows the metabolites that were significantly higher in the duodenum, jejunum, ileum, and liver of the MPF mice. It is notable that no other clusters contained features that were either unique to the GF or MPF subset of a tissue. After implementing PCA, volcano plots, and *k*-mean cluster analyses, the differential molecular features across all tissue types were taken into examination for metabolite identification. Supplementary Table 2 lists the annotated metabolites organized into their respective chemical classes and metabolic pathways for the thirteen tissues and their sub-groups (GF and MPF). Figures 4, 5, 6, 7 and 8 illustrate the annotated compounds across different metabolite classes in a heatmap chart, and will be discussed in following chapters.

### Microbiota affects various branches of amino acid and peptide metabolism

Our results show that majority of the amino acid metabolic pathways, including lysine, phenylalanine and tyrosine, polyamine, tryptophan, and arginine and proline (urea cycle) pathways, were affected by the presence or absence of the microbiota (Fig. 4). In lysine metabolic pathway, the level of lysine and 2-aminoadipic acid were higher in the GI tract tissues of GF mice than in their respective MPF group. In contrast, the rest of the identified metabolites, including *N*-acetyllysine, pipecolic acid, methyllysine, acetylhydroxylysine, carbamoylmethyllysine, glutarate, 5-acetamidovaleric acid, 5-aminovaleric acid betaine (5-AVAB), 2-piperidinone, and cadaverine, and showed lower abundance in the GF mice. Additionally, 2-aminoadipic and glutarate were also higher in the plasma of GF mice.

In phenylalanine and tyrosine metabolic pathway, the levels of phenylalanine, tyrosine, and *N*-acetylphenylalanine were significantly higher in the GI tract of GF mice than in their respective MPF counterparts. Other identified phenylalanine and tyrosine metabolites, *p*-cresol glucuronide, *p*-cresol sulfate, and 3-phenyllactic acid had lower abundance in the GF mice (Fig. 4). Notably, *p*-cresol glucuronide and *p*-cresol sulfate were found across all the examined tissue types, and as illustrated in the volcano plots (Fig. 3), they were also the most differential metabolites in multiple tissues; *p*-cresol glucuronide in plasma, heart, liver, pancreas, cecum, and colon, *p*-cresol sulfate in plasma, heart, liver, cecum, and SAT.

In tryptophan metabolic pathway (indole-containing compounds), tryptophan was significantly higher in abundance in the plasma, heart, muscle, cecum, and colon of GF mice (Fig. 4). The abundance of *N*-acetyltryptophan in the duodenum, cecum and colon of GF mice was also significantly higher than in their respective MPF counterparts. In contrast, abundance of tryptophan metabolites including 3-indoleacetic acid, 5-hydroxyindoleacetic acid, IPA, hydroxykynurenine, and oxindole were significantly lower in the cecum and colon of GF animals. Additionally, 5-hydroxyindoleacetic acid, IPA showed significantly low abundance in the plasma GF mice, and oxindole was significantly lower in all the GI tract tissues as well as in the pancreas and the liver.

All the identified metabolites from the polyamine metabolic pathway (*N*-acetylputrescine, *N*-carbamoylputrescine, *N*-acetylcadaverine, and diacetylspermidine) were observed throughout various examined tissues excluding muscle, and had a lower abundance in the GF group in most of the tissues (Fig. 4).

In the arginine and proline (urea cycle) metabolic pathways, the identified metabolites, urea, arginine, proline, homoproline, and carboxynorspermidine were markedly more abundant in the GI tract of GF mice than in that of the MPF mice, whereas only ornithine showed lower abundance in the cecum and colon of GF mice. Interestingly, BAT was the only tissue having a higher abundance of all the identified metabolites from this metabolic pathway in the GF mice (Fig. 4).

The results observed from the effect of the microbiota on peptide metabolism show a higher abundance of almost all the identified oligopeptides (di-, tri- and tetrapeptides) in the GI tract of GF mice (Fig. 5). However, peptides were not among the most significantly changed metabolites across the different tissues, with the exception of glutamylglutamic acid (Glu–Glu) and aspartyltyrosine (Asp–Tyr) that were the two peptides were also shown to be significantly higher in the cecum tissue of GF from volcano plots (based on the inclusion criteria from volcano plots with molecular features above the threshold (Fig. 3).

### Carbohydrates, products of energy and lipid metabolism are modified in response to the microbiota

The results herein indicated a higher level of mono- and disaccharides in the GF mice, particularly in the cecum and the colon (Fig. 6). The abundance of the identified metabolites, aconitic acid, citric acid, isocitric acid, and malate from energy metabolism, was higher in the GI tract tissues of GF mice. All of the mentioned metabolites were lower in abundance in the heart tissue and malate was absent in the heart of GF mice (Fig. 6). Notably, as also illustrated in the volcano plots (Fig. 3), malate was among the most differential compounds in the heart of MPF mice.

Bile acids were among the most differential metabolites affected by the gut microbiota, and they were found not only in the GI tract and liver, but throughout the examined tissues including heart, pancreas, and fat tissues (Fig. 7). GF animals had lower levels of identified primary bile acids, including chenodeoxycholic acid, allocholic acid, β-muricholic acid, norlithocholic acid, three isomers of hydroxy-oxo-cholan-24-oic acid, 7-HOCA (7alpha-hydroxy-3-oxo-4-cholestenoate), cholanic acid-diol-sulfoethyl-amide, and 7-sulfocholic acid. In contrast, the identified conjugated primary bile acids, taurocholic acid, and one of its isomers (i.e., taurallocholate, tauroursocholate, or taurohyocholate), tauro-α/β-muricholic acid, and several unidentified glycine and taurine-conjugated bile acids were higher in the GF mice (Fig. 7). The increase of taurine conjugated bile acids in the GF status was particularly evident in e.g., VAT tissue (Supplementary Fig. 1). The identified secondary bile acids, including 7-ketodeoxycholic acid, hyodeoxycholic acid, deoxycholic acid, taurochenodesoxycholic acid (taurochenodeoxycholic acid), taurodeoxycholic acid, lithocholic acid, and ursodeoxycholic acid were either completely missing from the GF mice or were present in negligible amounts.

In the carnitine and acylcarnitine metabolic pathways, carnitine, acetylcarnitine, clupanodonylcarnitine/adrenylcarnitine, malonylcarnitine, butyrylcarnitine, isovalerylcarnitine, linolenylcarnitine, and heptadecanoylcarnitine, showed lower abundance in the GI tract of GF mice, while hydroxyoctadecenoylcarnitine showed the higher abundance in the same mouse line compared to the MPF mice. Interestingly, l-carnitine had higher abundance in plasma samples of GF mice (Fig. 7). All the detected fatty amides were low in abundance in the GF mice in the cecum and the colon (Fig. 7).

### Flavonoids, phenolic acid derivatives, and terpenes were other chemical classes influenced by microbiota

The intestinal microbiota plays an important role in the metabolism of plant-derived phytochemicals including flavonoids, phenolic acid derivatives, and terpenes. The results herein indicate that the differences in identified flavonoids, phenolic acid derivatives, and terpenes between the GF and MPF mice were mostly found in the GI tract, and these differences were higher in or exclusive to the GF animals with a few exceptions’ high abundances in the MPF mice (Fig. 8). Exceptions included daidzein, 3-(3-hydroxyphenyl)propionic acid, dihydrocaffeic acid, gentisic acid, and medicagenic acid along with three unidentified flavonoids with molecular formulas C_18_H_18_O_8_ (two isomers) and C_21_H_22_O_10_ and one triterpenoid with formula C_30_H_48_O_2_ that were higher in GI tract of MPF mice. More specifically, gentisic acid, a bacterial end-metabolite of dietary (plant) salicylic acid^41^, along with microbial degradation products of the dietary phenolic acids, including 3-(3-hydroxyphenyl)propionic acid and dihydrocaffeic acid, were absent or existed in low amounts in the GF mice tissues. Various derivatives of the isoflavonoid compounds including daidzein and its sulfated form, formononetin and its glucuronide, genistein glucuronide, and genistein sulfate were also annotated in the data, and the compounds were differential in GI tract of the animals. Notably, as also illustrated in the volcano plots (Fig. 3), 3-(3-hydroxyphenyl)propionic acid was among the most differential metabolites in the cecum and the colon of MPF mice. Ferulic/isoferulic acid sulfate and vanillic acid sulfate were higher in the cecum of GF mice.

## Discussion

In this study, we observed how the presence or absence of gut microbiota has a strong influence on the biochemistry of mammalian tissues and revealed substantial variation in the distribution of metabolites from distinct chemical classes. Metabolite classes most affected were amino acids, peptides, carbohydrates, metabolic products of energy metabolism, lipids, particularly, bile acid, fatty amide, and acylcarnitine metabolism, and plant-derived phytochemicals, including flavonoids, phenolic acid derivatives, and terpenes. We herein also reported that all the GI tract tissues, as well as BAT and plasma, were the most affected tissues in the GF mice and showed higher percentage of significantly abundant metabolites when compared to their counterpart MPF mice in the same tissues. Finally, it is noteworthy that plasma was observed to have the lowest number of detectable metabolites compared to all other tissues.

The GF status of mice has an impact on the host metabolism^42^. This may suggest that with a lack of the gut microbiota and, subsequently, its related compounds in the GF mice, the host endogenous metabolism gives rise to different metabolic pathways that compensate for the loss or absence of microbiota. This hypothesis may also be supported by our observation of the high levels of mono- and disaccharides as well as energy metabolites in the GI tract of GF mice. It is already known that the gut microbiota modulates the energy balance of the host by allowing the host to harvest energy more efficiently from the digested food^43,44^. For example, carbohydrates undergo fermentation by gut bacteria that leads to the production of SCFAs, which serve as a major source of energy for the gut bacteria as well as host intestinal epithelial cells^45^. Since GF mice lack microbiota, we presume that the intestinal epithelial cells in this mouse group utilize sugars as the primary source of energy, thus exhibiting gluconeogenesis to synthesize more carbohydrate for the tricarboxylic acid (TCA) cycle. As a result, a higher abundance of mono- and disaccharides and aconitic acid, citric acid, isocitric acid, and malate from energy metabolic pathway in the GI tract of GF animals was observed when compared to their conventionally-raised counterparts. Similarly, in other previous metabolomics studies on GF and mice treated with antibiotic-induced microbiome depletion, significantly higher levels of monosaccharides and organic acids from the TCA cycle in the liver of GF mice were observed^46,47^. However, transcriptome profiling of several tissue from GF mice showed that GF mice are energy-deprived, and some of intermediate metabolites and enzymes of TCA cycle are significantly diminished^48,49^.

It is known that gut bacteria play an important role in host amino acid homeostasis^51–54^; once taken up by bacteria, amino acids can either be incorporated into bacterial cells for protein biosynthesis, become catabolized and used as an energy source, or get biotransformed to a diverse range of bioactive molecules, such as conversion of tryptophan to other indole-containing metabolites^51,55,56^. Our results showed a significant presence of peptides, mostly in the GI tract of GF mice. To our knowledge, there are no other studies showing similar large-scale differences in small peptide metabolism between germ-free and conventional mice via LC–MS metabolomics. Interestingly, data from transcriptomics studies of multiple tissues have also shown differential expression of long noncoding RNAs between germ-free and conventionally raised mice and these differences were highly tissue specific^57^. We speculate that high abundance of small peptides in GF mice is the result of higher expression of specialized ion-driven carrier proteins in the enterocyte to absorb nutrients more optimally ^58^.

Likewise, lysine, urea, proline, arginine, and the aromatic amino acids tryptophan, kynurenine, phenylalanine, and tyrosine with their acetylated forms, *N*-acetyltryptophan and *N*-acetylphenylalanine accumulated in the GI tract in the absence of gut microbiota. Through other studies including metagenomics and transcriptomics approaches, it is known that these metabolites serve as substrates for multiple pathways in gut microbiota (e.g., production of SCFAs)^35,55,56,59,60^. Thus, higher levels of these metabolites in the GI tract of GF mice may explain the critical role of gut microbiota in expanding the compounds diversity.

In alignment with previous findings^35,38,61^, we observed higher abundance of indoleacetic acid, hydroxyindoleacetic acid, IPA, and oxindole in MPF mice. These metabolites are microbial catabolites of tryptophan. Microbial catabolites of tryptophan are shown to have a potential role in the mediation of microbe-host interactions, and eventually contribute to the health status of the host^62^. Indoleacetic acid, hydroxyindoleacetic acid, and IPA are shown to regulate gut barrier function^62,63^ and merit further investigation for their potential role in reducing likelihood of cardiovascular diseases^64^ and developing type 2 diabetes^65,66^, likely by modulating host metabolism through the production of glucagon-like peptide-1 (GLP-1) to improve insulin resistance^62^. Oxindole, another tryptophan microbial metabolite, was higher in all the GI tract tissues, as well as in the liver and plasma in the MPF mice. High abundance of this metabolite is observed in impairment of insulin secretion in pancreatic beta cells and in hepatic encephalopathy conditions^67,68^, and merits further investigation for its potential role as a biomarker of hepatic cirrhosis.

In phenylalanine and tyrosine metabolic pathways, *p*-cresol sulfate, *p*-cresol glucuronide, and 3-phenyllactic acid are known metabolites of gut microbiota and lower levels of these metabolites in the GF mice are expected^38,69–71^. *p*-Cresol which is the precursor of *p*-cresol sulfate and *p*-cresol glucuronide, is one of the end-products of tyrosine and phenylalanine biotransformation by intestinal bacteria^72^. *p*-Cresol exerts many biological and biochemical toxic effects and should be excreted from the body^72^. In one of the mechanisms when this methylphenol metabolite reaches the mucosa of the colon^73^ and liver^74^, sulfatation and glucuronidation take place, as a result *p*-cresol sulfate and *p*-cresol glucuronide are generated. The two latter metabolites are less toxic and more water-soluble compared to the parent compound, *p*-cresol, which makes it easier for the body to get rid of them via urinary excretion.

In lysine pathway, lower levels of *N*-acetyllysine, pipecolic acid, methyllysine, acetylhydroxylysine, 5-acetamidovaleric acid, 5-AVAB, 2-piperidinone, cadaverine, and glutarate in the GF mice may be explained by the lack of gut microbiota, as the bacterial catabolism of lysine is shown to be one of the main contributing factors responsible for the production of these metabolites^75,76^. The other affected metabolite from this pathway was 2-aminoadipic acid. Although lysine was one of the main dietary constituents of both mouse groups in the study, the higher abundance of 2-aminoadipic acid, a metabolite of lysine catabolism, in plasma, ileum, cecum, and colon of the GF mice was observed. Studies showed that 2-aminoadipic acid induced higher energy expenditure in mice due to increased adipocyte thermogenesis^77^. It is also a regulator of glucose homeostasis^78^. These phenomena may be interrelated to the increase levels of mono- and disaccharides as well as energy metabolites observed in this study. However, we did not find any study reporting 2-aminoadipic acid in germ-free animals.

In polyamine metabolism, we observed lower levels of four polyamines, namely *N*-acetylputrescine, *N*-carbamoylputrescine, *N*-acetylcadaverine, and diacetylspermidine across many tissues. Polyamines are either synthesized endogenously or exogenously. Nevertheless, the exogenous source of polyamines (diet and gut microbiota) is the main contributor to the host polyamine pool^79,80^; a majority of the foodstuff contains polyamines and microbiota produces these compounds which are absorbed in great amounts in the large intestine^79^. Polyamines have various physiological functions and regulate multiple biological processes, including translation, transcription and cell proliferation and differentiation^80^, and, like SCFAs, are important intestinal growth factors that can contribute to the maintenance of intestinal homoeostasis^81^. To our knowledge, there are no other studies showing lower levels of polyamines across multiple tissues in germ-free mice compared to the conventionally-raised mice, and that may be explained either by the fact that GF mice may have altered gastrointestinal physiology^82^, or that the lack of bacterial-derived sources of polyamines may be the reasons why the low abundance of these compounds was observed in the GF mice in our study^83^.

**Figure 9:**
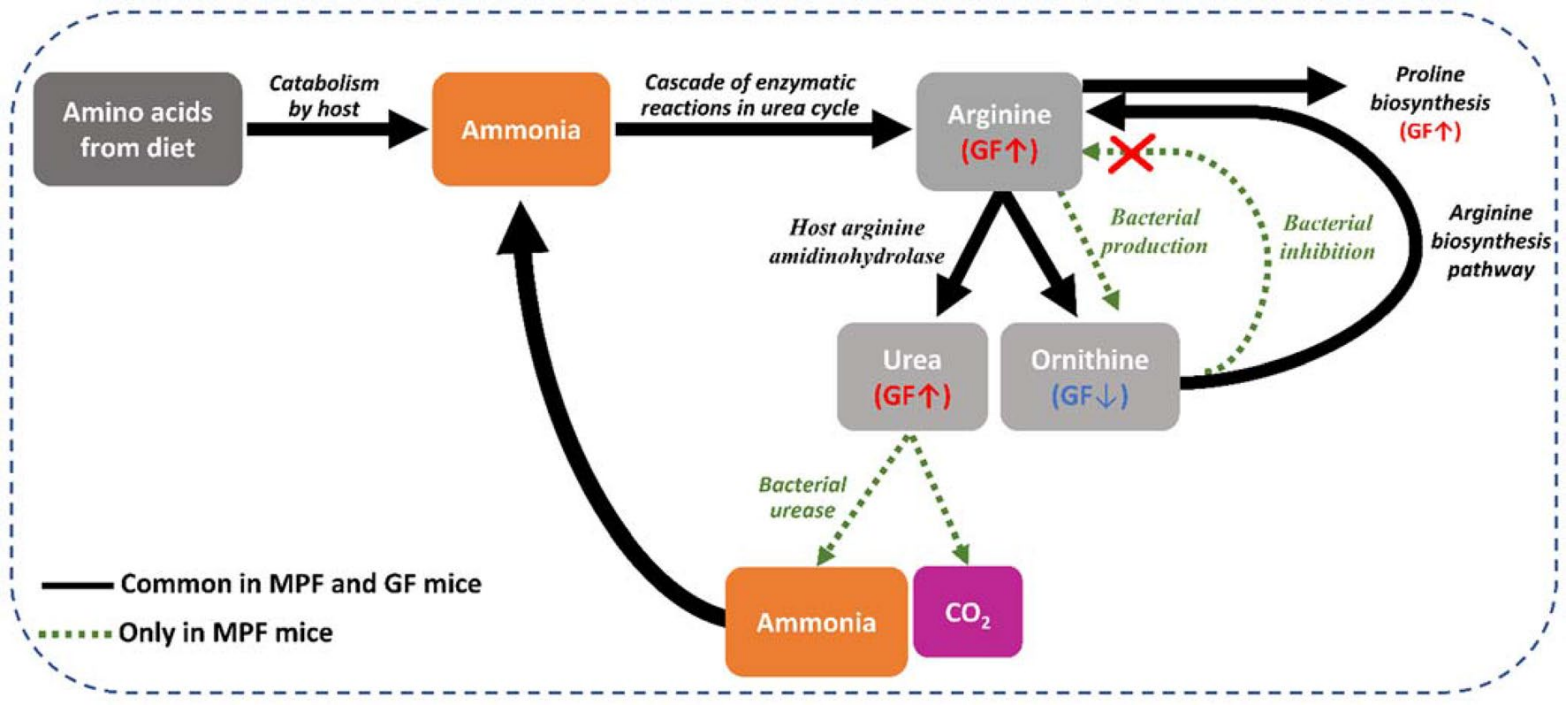
Summary of the fate of key metabolites (arginine, proline, urea, and ornithine) in the arginine and proline metabolism (urea cycle). Catabolism of dietary amino acids leads to the production of ammonia. Ammonia further undergoes conversion to urea via the urea cycle. In mammals with conventional gut microbiota, urea is broken down to ammonia and CO_2_ by bacterial urease. The ammonia produced by microbiota is released into the GI tract and is taken up by host cells and serves as a substrate to synthesize arginine in the urea cycle. Within the urea cycle, arginine is then converted into urea and ornithine. Simultaneously, ornithine can also be synthesized by gut bacteria. Ornithine produced from the two mentioned pathways, can enter the arginine biosynthesis pathway to synthesize more arginine; nevertheless, the bacterial inhibition of arginine biosynthesis can be inhibited by some bacteria. This excess amount of arginine can either enter the arginine and proline biosynthesis pathways or the urea cycle.

In arginine and proline metabolism (urea cycle), we observed elevated level of urea in the GF mice, particularly, in the cecum and colon. In a recent study by Pessa-Morikawa et al., similar trend was reported in intestine, brain and placental tissues^39^. Earlier studies suggest that the level of intestinal urea was decreased in conventionally-raised mice through the generation of ammonia^83^. The underlying mechanism is yet to be elucidated however, we speculate that the elevated levels of urea in the GF mice is most likely due to breakdown of urea by gut microbiota urease and its bioconversion into ammonia and carbon dioxide (Fig. 9)^84^. This proposed mechanism may also be the reason for higher levels of arginine and proline in GF mice. Lower levels of ornithine in the same mouse group could be explained by the promotion of bacterial ornithine production from arginine, which accumulated throughout the majority of tissues examined from GF mice^85^, as well as the bacterial inhibition of arginine biosynthesis from ornithine^86^. Higher abundance of carboxynorspermidine, another metabolite of the arginine and proline metabolism (urea cycle), in GF mice was observed. Carboxynorspermidine is a bacterial substrate for norspermidine biosynthesis^87^ and elevated levels of this metabolite may be explained by lack of microbiota in this mouse group to convert carboxynorspermidine to norspermidine^87^.

Among the lipid chemical subclasses, the metabolic pathways that were significantly altered included bile acid metabolism, fatty amide metabolism, and fatty acid (acylcarnitine) metabolism. Bile acids have gained special attention in their relation to gut microbiota because of secondary bile acids, which have a microbial origin^88^. In our study, the bile acids were among the most widely distributed metabolites across the different tissue types, which reflect a potentially-significant role in metabolism of different organs^89^. Despite the profound modulatory impact of gut microbiota on bile acids (i.e., deconjugation, dehydrogenation, and dihydroxylation of primary bile acids), bile acid metabolism that occurs in the germ-free mice is rather complex. Previous studies have indicated high variability in bile acid metabolism between germ-free and conventional mouse models^90^; the phenotypes conceivably depend on diet, sex, genetic background, and the respective composition of the microbiota in the conventional control groups^90,91^. The results herein showed lower levels of primary bile acids in the GI tract of GF mice with a few exceptions in tauro-conjugated primary bile acids which had a higher abundance in this mouse group (i.e., tauro-α/β-muricholic acid, taurocholic acid, and an isomer of taurocholic acid). Mice lacking intestinal bacteria have no deconjugation effect on the amino acid moiety of conjugated primary bile acids, and as they pass through the GI tract, they remain intact^92^. Aligned with this fact, higher abundance of tauro-conjugated primary bile acids in our study may be explained. On the other hand, all the identified secondary bile acids were missing from the GF mice, confirming that gut microbiota was absent in these mice.

**Figure 10:**
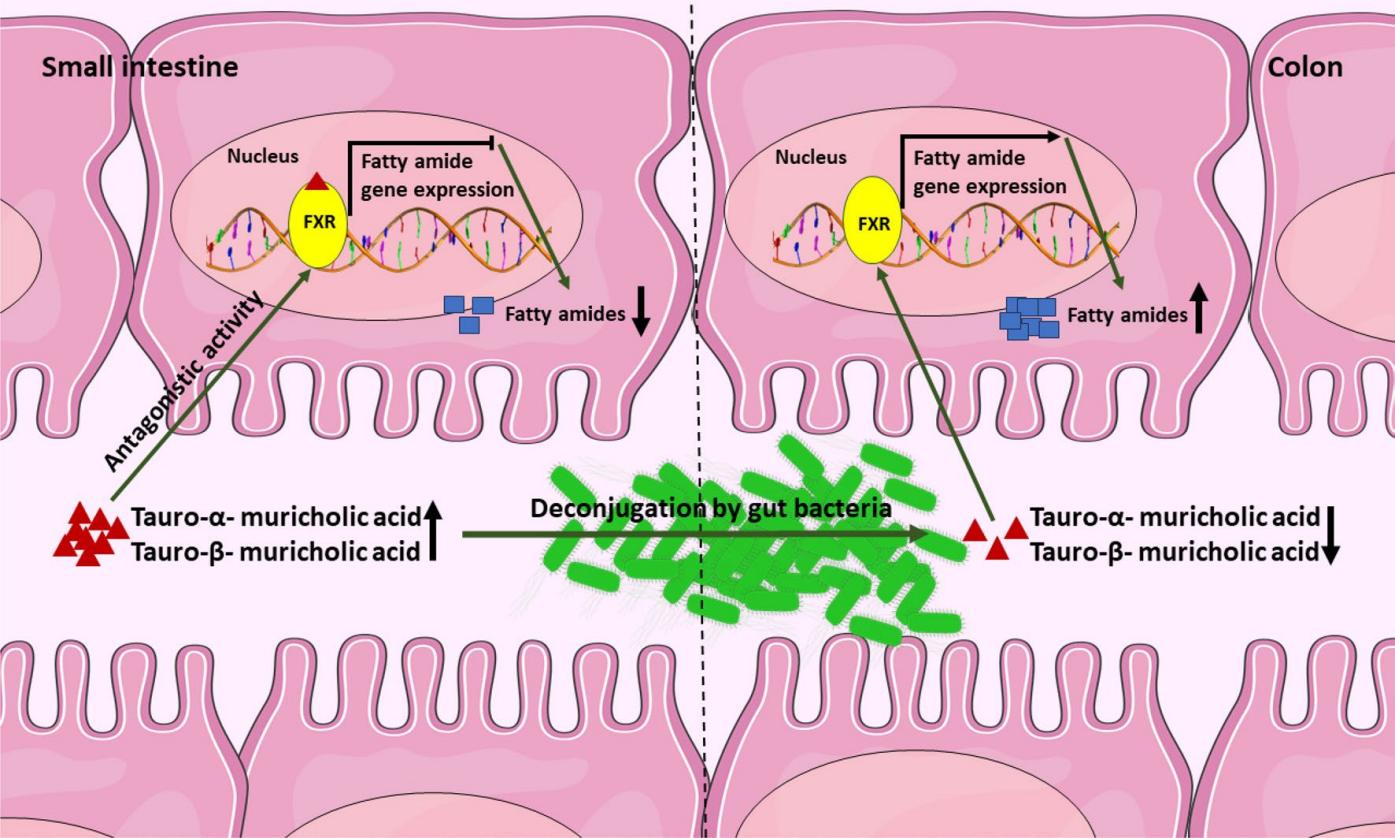
Proposed interrelation between tauro-conjugated muricholic acids and fatty amide biosynthesis in proximal and distal GI tract. When the FXR is bound to tauro-α- and β-muricholic acids in the upper section of the GI tract, the fatty amides biosynthesis is downregulated. As tauro-α- and -β-muricholic acids pass through the GI tract, they get deconjugated by gut microbiota. Therefore, there are fewer tauro-α- and β-muricholic acids are available to bind to FXR in the lower section of the GI tract. Thus, the FXR expression is upregulated in this area, and that may explain the higher abundances of fatty amides in the lower part of the GI tract.

Another lipid subclass with a significant difference between the two mouse lines was fatty amides. To our knowledge, there are no other studies comparing the levels of fatty amides between GF and MPF mice. In our data, the MPF mice showed lower abundance of fatty amides in the upper part of the GI tract compared to the lower part. Conversely, we observed a higher abundance of tauro-conjugated bile acids in the upper part of the GI tract when compared to the lower part of the GI tract of the same mouse group. Based on this observation, we propose a mechanism in which fatty amide metabolism may be interrelated with bile acid metabolism; some bile acids, including tauro-α- and β-muricholic acids (in mice) and ursodeoxycholic acid (in humans) are known to be potent antagonists of the bile-acid-activated nuclear receptor, farnesoid X receptor (FXR)^17,93^. Additionally, in a study reported by Gonzalez et al., it was shown that FXR could be bound by a number of endogenous bile acids, including tauro-α- and β-muricholic acids in the upper section of the GI tract expected^94^. Given this fact, we herein speculate that because of this affinity between FXR and tauro-conjugated-muricholic acids, the fatty amides gene expression towards their biosynthesis is downregulated in the upper part of the GI tract of MPF mice. The low abundance of tauro-α- and β-muricholic acids in the lower part of the GI tract of the same mouse group may be justified by the gut microbiota enzymatic activity through deconjugation as they pass through the GI tract^94^. Therefore, fewer tauro-α- and β-muricholic acids are available to bind to FXR in the lower section of the GI tract. As a result, the FXR expression and fatty amide biosynthesis are upregulated in this area, and that may explain the higher abundances of fatty amides in the lower part of the GI tract of MPF mice (Fig. 10). However, more experimentations such as gene expression analysis in conventional and germ-free mice are required to fully validate this proposed model.

Acylcarnitines are essential for oxidative catabolism of fatty acids as well as energy homeostasis in the host. Their accumulation in different organs is an indication of incomplete mitochondrial fatty acid β-oxidation and are important diagnostic markers of mitochondrial fatty acid β-oxidation disorders^95,96^. We observed higher levels of acylcarnitines in tissues of MPF mice. In a recent study by Smith et al. (2021), the authors showed higher fecal levels of acylcarnitines in GF mice compared with conventionally-raised mice^95^. Having provided evidence, we postulate that the intestinal epithelial cells in MPF mice are more capable of absorbing acylcarnitines from the gut lumen and utilize for their mitochondrial oxidative metabolism, compared to their germ-free counterparts. Additionally, as part of their metabolic fate, some fatty acids are converted to intermediate metabolites by gut bacteria. These bacterial fatty acid intermediates can further be absorbed and metabolized through β-oxidation in the host mitochondria, which can then be converted into acylcarnitines and excreted (Supplementary Fig. 3)^97^. This phenomenon may account for the role of gut microbiota in inducing acylcarnitine production in the MPF mice, as it was observed in this study, and may be an indication of diminished fatty acid oxidation in germ-free mice. Amongst the acylcarnitines that were detected in the plasma, l-carnitine was the only metabolite that was not detected in plasma of MPF mice. Concurrent with our observation, Lai et al., also showed a similar trend from fecal metabolic profiling of germ-free mice^38^. Aside from its main source, diet, L-carnitine is synthesized endogenously in liver and kidney from two essential amino acids, lysine and methionine. On the other hand, l-carnitine is metabolized by gut bacteria and liver to produce TMAO^98^. Nonetheless, the responsible biochemical pathway(s) and required genetic components in gut bacteria to metabolize l-carnitine to TMAO is not yet fully understood^98,99^. TMAO was our reference metabolite to assure the germ-free status of the mice used in the study, as it is a metabolite with microbial origin^100^. Besides the slow turnover of carnitine in the body^101^, we speculate that the absence of microbiota and lysine supplementation in the diet (NIH #31M Rodent Diet) may account for higher plasma level of l-carnitine in the GF mice. As mentioned, with the absence of microbiota, the GF mice are not able to convert carnitine to TMAO, and with additional lysine supplementation, this amino acid can enter the carnitine biosynthesis pathway to produce more carnitine.

We observed higher levels of flavonoids, phenolic acid derivatives, and terpenes in the GF mice and interestingly, these secondary metabolites were mostly present in the GI tract of the animals. NIH #31M Rodent Diet, used as animal feed in this study, comprises soybean and whole grains (wheat, oats, and corn). Therefore, it is likely that the diet contains a high number of phytochemicals that consequently reflect the mice tissue metabolome. Most of these compounds have very low bioavailability^102^. We postulate that when the phenolic compounds and terpenes travel through the GI tract of germ-free mice, unlike the MPF mice, the lack of resident gut microbiota in these mice hinders their catabolism or biotransformation into the smaller phenolic metabolites that are more easily absorbed and could exhibit enhanced bioavailability compared to their parent compounds. Examples from this study include a higher abundance of daidzein and 3-(3-hydroxyphenyl)propionic acid in the majority of the tissues as well as dihydrocaffeic acid, gentisic acid, and medicagenic acid along with three unidentified flavonoids with molecular formulas C_18_H_18_O_8_ (two isomers) and C_21_H_22_O_10_ and one triterpenoid with formula C_30_H_48_O_2_ in the cecum and the colon in MPF mice. Daidzein, the aglycone form of daidzin, is one of the widely studied isoflavones from soybeans. Following ingestion, daidzin is hydrolyzed by gut microbial glucosidases (some strains of *Bifidobacterium*, *Lactobacillus*, *Lactococcus,* and *Enterococcus),* which release the more bioavailable and potent metabolite against oxidative stress and cancer, daidzein^103–105^. 3-(3-Hydroxyphenyl)propionic acid and dihydrocaffeic acid are respectively the dehydroxylated and hydrogenated forms of caffeic acid (a common dietary component found in a variety of plant-derived food products) produced by gut microbiota (*Bifidobacterium*, *Escherichia*, *Lactobacillus*, and *Clostridium*) and merit attention for their antioxidant activities^106–109^. Gentisic acid, an active microbial metabolite of salicylic acid hydroxylation, is shown to inhibit colorectal cancer cell growth^41^. On the other hand, a higher abundance of ferulic/isoferulic acid sulfate and vanillic acid sulfate in the majority of the GF mice tissues was observed. We speculate that, as their parent compounds, ferulic/isoferulic acid and vanillic acid, did not undergo the microbial biotransformation, they were absorbed intact, reaching the liver to be converted into the sulfated form^110^. In general, while the characteristics of most of the identified phytochemicals and their derivatives that existed in higher abundance in the GF mice cannot be assessed in this study, it can be hypothesized that the presence of microbiota is essential in modulating the pool of phytochemicals entering the host tissues and exerting their physiological effect.

### Study limitations

The important study limitations to consider in these outcomes was that with the current MS/MS databases, only a relatively small portion of the metabolites can be identified or putatively annotated. Additionally, in this study, the number of molecular features detected, especially in the tissue samples, was remarkably high, and therefore the identification could only be focused on the fraction of the most differential features within each tissue. In the current study, we report the discovery of several microbiota-related metabolite findings in an organ-dependent manner, thus, validation of these findings in other separate cohorts and in other germ-free mouse models is warranted. It is evident that the germ-free status caused massive differences across all the examined tissues and should therefore be addressed in a tissue-specific manner, alongside with other analytical techniques e.g., gene expression analysis. Models including germ-free animal studies have been widely used as a source of knowledge on the gut microbiota contributions to host homeostatic controls as they allow disruption in the gut microbiota to be studied in a controlled experimental setup^100^. However, when translating the results from gut microbiome research from mouse models to humans, there are pitfalls to be considered as these two species are different from another in anatomy, genetics and physiology^111^, and there are no animal models perfectly replicate clinical conditions. Additionally, germ-free animals may not represent the best translational model for studying the functionality of the microbiota since they have intrinsically underdeveloped immunological responses, shortened microvilli, and many other structural and functional differences compared to conventional animals. However, studies showed that germ-free animals are valid in vivo experimental models for preclinical studies and for investigating the host-microbial interactions in health and diseases^112^. Nevertheless, further investigations are needed to understand the massive impact of the microbiota on biochemicals at the intersection of food and human metabolism, and eventually, on health.

## Conclusions

In summary, we have demonstrated a significant effect of the microbiome on the metabolic profile of 13 different murine tissues by applying a non-targeted metabolomics approach to a germ-free mouse model system. The results support the hypothesis that the chemistry of all major tissues and tissue systems are affected by the presence or absence of a microbiome. The strongest signatures come from the gut through the modification of host amino acids and peptides, carbohydrates, energy metabolism, bile acids, acylcarnitines, fatty amides, and xenobiotics, particularly the breakdown of plant-based natural products, flavonoids, and terpenes from food. Noteworthily, we also showed large scale differences in small peptide metabolism, fatty amides, and polyamines across multiple tissues in germ-free mice compared to the conventionally-raised mice that to our knowledge, no other studies have addressed yet. Growing evidence from animal models and human studies supports that the microbiota is a key to various aspects of our health. As the link between humans and their microbial symbionts becomes more and more apparent, a combination of global non-targeted approaches and the development of tools that connect these data sets warrants greater attention to enable us to identify novel metabolites, leading to a better understanding of the deep metabolic connection between our microbiota and our health—with diet in between. We propose further investigations to uncover unique, organ-specific microbial signatures, and whether the specific inter-organ microbial signature can be linked to the host metabolic diseases such as obesity and its related disorders.

## Methods

### Tissue sample collection and preparation

Blood, heart, liver, pancreas, muscle, duodenum, jejunum, ileum, cecum, colon, VAT, SAT, and BAT tissues from five GF and five MPF male C57BL/6NTac mice age 10 weeks were obtained from Taconic Biosciences (www.taconic.com/mouse-model/black-6-b6ntac). All animal experiments complied with the ARRIVE guidelines. All procedures were approved under animal care and use protocol number IC0033, Taconic In-Vivo Scientific Services Blanket Protocol and were performed in accordance with relevant guidelines and regulations. Taconic’s animal care and use programs are fully AAALAC accredited; US programs are also PHS Assured (D16-00,323; legacy no. A3524-01). Where applicable, IACUC approved protocols or government approved licenses govern animal use in research, testing and teaching applications; including production and use of genetically engineered mouse and rat models. Mice were maintained on a standard circadian cycle of 12:12 h light:dark cycle. The lights would turn on around 5:00 AM and turn off around 17:00. The mice were sacrificed after ~ 5 h of light, and the organ samples were collected immediately. The sterile natural ingredient NIH #31 M Rodent Diet (www.taconic.com/quality/animal-diet) was used as the standard diet. To assure the germ-free status of the mice used in the study, TMAO, a metabolite with microbial origin^75,100^, was used as a reference compound. Blood was collected (K2-EDTA Microtainer Tubes) and centrifuged at 3000*g* for 10 min at room temperature. Other tissues were rinsed with phosphate-buffered saline thoroughly. Furthermore, all the tissues were snap-frozen in liquid nitrogen and shipped on dry ice. Upon arrival, samples were immediately transferred and kept at − 80 °C until further processing for metabolomics.

All frozen samples were cryo-ground and then 100 ± 2 mg of powdered sample was cryo-weighted into 1.5 mL Eppendorf tubes. The tissues (except plasma) were treated with 80% ice-cold methanol in a ratio of 300 µL solvent per 100 mg tissue. Then the samples were briefly vortexed and incubated on a shaker (Heidolph Multi Reax) at 2000 rpm for 15 min at room temperature. For plasma samples, acetonitrile (ACN) was used as a solvent with the ratio of 1:4 vol:vol (plasma to solvent) and vortexed. All the samples were centrifuged for 10 min at 4 °C (18,000*g*), and the supernatant fractions were filtered using 0.2-µm Acrodisc Syringe Filters with a PTFE membrane (PALL Corporation) and stored at − 20 °C until further analysis with LC–MS. The order of the samples was randomized before the analysis.

### Instrumentation

We used ultra-high-performance liquid chromatography (1290 LC system, Agilent Technologies, Santa Clara, CA) with high-resolution mass spectrometry (6540 Q-TOF-MS, Agilent Technologies, Santa Clara, CA) for non-targeted metabolite profiling^113–115^. We used two chromatographic separation techniques, which were hydrophilic interaction chromatography (HILIC) and reversed-phase (RP), and further acquired data in both positive and negative electrospray ionization (ESI) modes, optimized for as wide metabolite coverage as possible. Aliquots of 2 μL from all the specific sample matrices were generated as a pooled quality control sample (QC) and were injected in the beginning of the analysis as well as between sample types (every 10th injection). For RP analysis, the mobile phase flow rate was 500 μL/min with Zorbax RRHD Eclipse XDB-C18 column (100 × 2.1 mm, 1.8 μm; Agilent Technologies). The column temperature was maintained at 50 °C. Mobile phase was 0.1% v/v formic acid in water (A) and 0.1% (v/v) formic acid in methanol (B). We used gradient elution which was as follows: 0–10 min: 2% → 100% solution B; 10–14.5 min: 100% solution B; 14.5–14.51 min: 100% → 2% solution B; and 14.51–16.5 min: 2% solution B. For HILIC analysis, mobile phase flow rate was 600 µL/min with Acquity UPLC BEH Amide column (100 mm × 2.1 mm, 1.7 μm; Waters Corporation, Milford, MA). The column temperature was maintained at 45 °C. Mobile phase was 50% v:v acetonitrile (A) and 90% v:v acetonitrile (B). Both solvents contained 20 mmol/L ammonium formate, pH 3. The following gradient elution was used: 0–2.5 min, 100% B; 2.5–10 min, 100% B → 0% B; 10–10.1 min, 0% B → 100% B; 10.1–14 min, 100% B. The sample injection volume was 3 μL and the sample tray temperature was kept at 4 °C during the analysis.

The mass spectrometry (MS) conditions were: drying gas temperature of 325 °C with a flow of 10 L/min, a sheath gas temperature of 350 °C and a flow of 11 L/min, a nebulizer pressure of 45 psi (310 kPa), capillary voltage of 3,500 V, nozzle voltage of 1000 V, fragmentor voltage of 100 V, and a skimmer voltage of 45 V. Data acquisition was performed using extended dynamic range mode (2 GHz), and the instrument was set to acquire ions over the mass range *m/z* 50–1,600. Data were collected in the centroid mode at an acquisition rate of 2.5 spectra/s (i.e., 400 ms/spectrum) with an abundance threshold of 150. For automatic data-dependent MS/MS analyses, the precursor isolation width was 1.3 Da, and from every precursor scan cycle, the 4 ions with the highest abundance were selected for fragmentation with the collision energies of 10, 20, and 40 V. These ions were excluded after 2 product ion spectra and released again for fragmentation after a 0.25 min hold. The precursor scan time was based on ion intensity, ending at 20,000 counts or after 300 ms. The product ion scan time was 300 ms. Continuous mass axis calibration was applied throughout the analysis using two reference ions *m/z* 121.050873 and *m/z* 922.009798 in the positive mode and *m/z* 112.985587 and *m/z* 966.000725 in the negative mode.

### Data extraction and compound identification

Raw data was processed through MS-DIAL software (version 3.00) for baseline filtering, baseline calibration, peak picking, identification, peak alignment, and peak height integration^116^. Centroid spectra peaks higher than 400 counts were restricted to ion species [M−H]^−^ and [M+Cl]^−^ in negative and [M+H]^+^ and [M+Na]^+^ in positive modes. The mass tolerance for compound mass was ± 15 mDa, retention time ± 0.2 min, and symmetric expansion value ± 10 mDa for chromatograms. Compounds were identified by comparison to library entries of purified standards and compared against METLIN (https://metlin.scripps.edu), MassBank of North America (MoNA, https://mona.fiehnlab.ucdavis.edu), Human Metabolome Database (HMDB, www.hmdb.ca), and LIPID MAPS (www.lipidmaps.org) metabolomics databases. The MS/MS fragmentation of the metabolites was compared with candidate molecules found in databases and verified with earlier literature on the same or similar compounds. Metabolomics Center of Biocenter Kuopio maintains an in-house library of over 600 authenticated standards that contains the retention time, mass to charge ratio (*m/z*), and chromatographic data (including MS/MS spectral data) on all molecules present in the library.

### Statistical analysis

The combined data matrix, i.e., HILIC (positive and negative ionization modes) and RP (positive and negative ionization modes) comprised 24,294 molecular features from 13 tissues from the GF and MPF mice, which underwent statistical analysis. There was a total of 5961 and 3946 molecular features obtained from HILIC, and 9256 and 5131 features from RP, in positive and negative ionization modes, respectively. Before performing any statistical analysis, the false zero values were imputed for each mouse group in a tissue, individually. An arbitrary raw abundance value of 10,000 was set as the threshold. Following rules were applied based on the signals of biological replicate measurements: (1) if the metabolite raw abundance was zero in more than 60% of the replicates, then the zero values were considered true zero. Therefore, all the non-zero values were replaced with zero regardless if they were smaller or higher than the arbitrary threshold (i.e*.*, default intensity of 10,000); (2) if the metabolite raw abundance was zero in less than 60% of the replicates and the non-zero values were lower than the threshold, then zero values were also considered true zero and the non-zero values were replaced with zero; (3) if the metabolite raw abundance was zero in less than 60% of the replicates and the non-zero values were higher than the threshold, then zero values were considered false and were replaced with an imputed value. The imputed value was calculated for each molecular feature as the average of the non-zero raw abundances in that tissue-specific mouse group.

A fold change (FC) value was calculated for each molecular feature and tissue by dividing the average signal abundance of the GF samples (“treatment” group) with that of the MPF samples (“control” group). Thus, FC > 1 signifies a higher abundance of the molecular feature in the GF mice and FC < 1 a higher abundance in the MPF mice.

Data processing was carried out by R package (3.5.3) for unscaled data. Mann–Whitney *U-*test was chosen to identify the most differentially abundant molecular features between the GF and MPF for the tissue-specific metabolite levels. False discovery rate (FDR, corrected *p* value, *q* value) was performed based on Benjamini and Hochberg's method. Significant metabolites had *p* values of ≤ 0.05 and *q* values below the threshold of ≤ 0.05.

The raw abundances of metabolites were first z-normalized based on the following formula: x_normalized_ = (x − $\overline{x }$_row_)/SD_row_. The *k*-means cluster analysis and hierarchical clustering Figures 4, 5, 6, 7 and 8, Supplementary Figure S2) were performed by the open-source software Multi experiment Viewer (MeV, http://mev.tm4.org) to visualize the common trends in the profile of the different molecular features (*k*-means clustering) as well as visualizing the abundance of the features when compared against the other molecular features present in the same cluster. The number of clusters was set to 10. The number of clusters was optimized based on visual inspection to reveal as many clusters as possible with a distinct pattern of molecular features without having two similar clusters.

After the statistical analysis, further filtering was applied on the obtained differential metabolic features based on the following inclusion criteria. The inclusion criteria must have existed in at least one tissue and one mouse group (i.e., GF or MPF): (1) fold change ≥ 1.3 with a *p* value ≤ 0.05, and *q*-value ≤ 0.05 (2) high-intensity metabolite values (raw abundance ≥ 100,000), (3) containment of MS/MS fragmentation, (4) and retention time ≥ 0.7 min. This filtering procedure resulted in a set of 4605 statistically significant molecular features, which then underwent *k*-means clustering analysis. Principal component analysis (PCA) was applied to all the 24,294 molecular features for visualization of the overall metabolite feature pattern of different tissues. Tissue-wise volcano plot visualization was applied on the statistically significant molecular features to display discriminatory molecular features within a tissue between the GF and MPF mice using MetaboAnalyst platform (https://www.metaboanalyst.ca/)^117^.

### Ethics approval and consent to participate

The animal experimental part was performed by Taconic Biosciences who is licensed to ethical animal care and use.

### Supplementary Information

Supplementary Information 1.

Supplementary Information 2.

Supplementary Information 3.

Supplementary Information 4.

Supplementary Information 5.

## Data Availability

The datasets supporting the conclusions of this article are available as supplementary material alongside this publication.

## Abbreviations

GF: Germ-free
MPF: Murine-pathogen-free/conventional
GI: Gastrointestinal
MAC: Microbiota-accessible carbohydrate
SCFA: Short-chain fatty acid
IBD: Inflammatory bowel disease
VAT: Visceral adipose tissue
SAT: Subcutaneous adipose tissue
BAT: Brown adipose tissue
TMAO: Trimethylamine N-oxide
CAN: Acetonitrile
HILIC: Hydrophilic interaction chromatography
RP: Reversed-phase
ESI: Electrospray ionization
QC: Quality control
LC–MS: Liquid chromatography-mass spectrometry
MoNA: MassBank of North America
HMDB: Human metabolome database
FC: Fold change
FDR: False discovery rate
MeV: Multi experiment viewer
PCA: Principal component analysis
5-AVAB: 5-Aminovaleric acid betaine
Glu-Glu: Glutamylglutamic acid
Asp-Tyr: Aspartyltyrosine
7-HOCA: 7Alpha-hydroxy-3-oxo-4-cholestenoate
GLP-1: Glucagon-like peptide-1
FXR: Farnesoid X receptor

## References

[1] Sidhu M, van der Poorten D (2017). The gut microbiome. Austral. Family Physician.

[2] Berg G (2020). Microbiome definition re-visited: Old concepts and new challenges. Microbiome.

[3] Marchesi JR, Ravel J (2015). The vocabulary of microbiome research: A proposal. Microbiome.

[4] Huttenhower C (2012). Structure, function and diversity of the healthy human microbiome. Nature.

[5] Wang B, Yao M, Lv L, Ling Z, Li L (2017). The human microbiota in health and disease. Engineering.

[6] Gilbert JA (2018). Current understanding of the human microbiome. Nat. Med..

[7] Hehemann JH (2010). Transfer of carbohydrate-active enzymes from marine bacteria to Japanese gut microbiota. Nature.

[8] Turnbaugh PJ, Gordon JI (2009). The core gut microbiome, energy balance and obesity. J. Physiol..

[9] Sonnenburg ED (2016). Diet-induced extinctions in the gut microbiota compound over generations. Nature.

[10] McNeil NI (1984). The contribution of the large intestine to energy supplies in man. Am. J. Clin. Nutr..

[11] Duncan SH (2007). Reduced dietary intake of carbohydrates by obese subjects results in decreased concentrations of butyrate and butyrate-producing bacteria in feces. Appl. Environ. Microbiol..

[12] Shafquat A, Joice R, Simmons SL, Huttenhower C (2014). Functional and phylogenetic assembly of microbial communities in the human microbiome. Trends Microbiol..

[13] Johnson EL (2019). Sphingolipid production by gut Bacteroidetes regulates glucose homeostasis. bioRxiv.

[14] Quinn RA (2019). Chemical impacts of the microbiome across scales reveal novel conjugated bile acids. bioRxiv.

[15] Ramirez-Perez O, Cruz-Ramon V, Chinchilla-Lopez P, Mendez-Sanchez N (2017). The role of the gut microbiota in bile acid metabolism. Ann. Hepatol..

[16] De Vadder F (2014). Microbiota-generated metabolites promote metabolic benefits via gut-brain neural circuits. Cell.

[17] Sayin SI (2013). Gut microbiota regulates bile acid metabolism by reducing the levels of tauro-beta-muricholic acid, a naturally occurring FXR antagonist. Cell Metab..

[18] Brestoff JR, Artis D (2013). Commensal bacteria at the interface of host metabolism and the immune system. Nat. Immunol..

[19] Kamada N, Chen GY, Inohara N, Nunez G (2013). Control of pathogens and pathobionts by the gut microbiota. Nat. Immunol..

[20] Abreu MT (2010). Toll-like receptor signalling in the intestinal epithelium: How bacterial recognition shapes intestinal function. Nat. Rev. Immunol..

[21] Claus SP, Guillou H, Ellero-Simatos S (2016). The gut microbiota: A major player in the toxicity of environmental pollutants?. NPJ Biofilms Microbiomes.

[22] Sousa T (2008). The gastrointestinal microbiota as a site for the biotransformation of drugs. Int. J. Pharm..

[23] Johnson KV, Foster KR (2018). Why does the microbiome affect behaviour?. Nat. Rev. Microbiol..

[24] Dinan TG, Cryan JF (2015). The impact of gut microbiota on brain and behaviour: Implications for psychiatry. Curr. Opin. Clin. Nutr. Metab. Care.

[25] Long-Smith C (2020). Microbiota-gut-brain axis: New therapeutic opportunities. Annu. Rev. Pharmacol. Toxicol..

[26] Hall AB, Tolonen AC, Xavier RJ (2017). Human genetic variation and the gut microbiome in disease. Nat. Rev. Genet..

[27] Spor A, Koren O, Ley R (2011). Unravelling the effects of the environment and host genotype on the gut microbiome. Nat. Rev. Microbiol..

[28] Yatsunenko T (2012). Human gut microbiome viewed across age and geography. Nature.

[29] Zhang C (2010). Interactions between gut microbiota, host genetics and diet relevant to development of metabolic syndromes in mice. ISME J..

[30] Landberg R, Hanhineva K (2019). Biomarkers of a healthy nordic diet-from dietary exposure biomarkers to microbiota signatures in the metabolome. Nutrients.

[31] Clemente JC, Ursell LK, Parfrey LW, Knight R (2012). The impact of the gut microbiota on human health: An integrative view. Cell.

[32] Holmes E, Li JV, Athanasiou T, Ashrafian H, Nicholson JK (2011). Understanding the role of gut microbiome-host metabolic signal disruption in health and disease. Trends Microbiol..

[33] Jeffery IB, Lynch DB, O'Toole PW (2016). Composition and temporal stability of the gut microbiota in older persons. ISME J..

[34] Reimer RA (2019). Establishing the role of diet in the microbiota–disease axis. Nat. Rev. Gastroenterol. Hepatol..

[35] Wikoff WR (2009). Metabolomics analysis reveals large effects of gut microflora on mammalian blood metabolites. Proc. Natl. Acad. Sci. USA.

[36] Vernocchi P, Del Chierico F, Putignani L (2016). Gut microbiota profiling: Metabolomics based approach to unravel compounds affecting human health. Front. Microbiol..

[37] Velagapudi VR (2010). The gut microbiota modulates host energy and lipid metabolism in mice. J. Lipid Res..

[38] Lai Y (2021). High-coverage metabolomics uncovers microbiota-driven biochemical landscape of interorgan transport and gut-brain communication in mice. Nat. Commun..

[39] Pessa-Morikawa T (2022). Maternal microbiota-derived metabolic profile in fetal murine intestine, brain and placenta. BMC Microbiol..

[40] Marcobal A (2013). A metabolomic view of how the human gut microbiota impacts the host metabolome using humanized and gnotobiotic mice. ISME J..

[41] Sankaranarayanan R (2020). Aspirin metabolites 2,3-DHBA and 2,5-DHBA inhibit cancer cell growth: Implications in colorectal cancer prevention. Mol. Med. Rep..

[42] Wang X (2020). Aberrant gut microbiota alters host metabolome and impacts renal failure in humans and rodents. Gut.

[43] Backhed F (2004). The gut microbiota as an environmental factor that regulates fat storage. Proc. Natl. Acad. Sci. USA.

[44] Heiss CN, Olofsson LE (2018). Gut microbiota-dependent modulation of energy metabolism. J. Innate Immun..

[45] Morrison DJ, Preston T (2016). Formation of short chain fatty acids by the gut microbiota and their impact on human metabolism. Gut Microbes.

[46] Chuang H-L (2012). Metabolomics characterization of energy metabolism reveals glycogen accumulation in gut-microbiota-lacking mice. J. Nutr. Biochem..

[47] Zarrinpar A (2018). Antibiotic-induced microbiome depletion alters metabolic homeostasis by affecting gut signaling and colonic metabolism. Nat. Commun..

[48] Donohoe DR (2011). The microbiome and butyrate regulate energy metabolism and autophagy in the mammalian colon. Cell Metab..

[49] Gnainsky Y (2021). Systemic regulation of host energy and oogenesis by microbiome-derived mitochondrial coenzymes. Cell Rep..

[50] Portune KJ (2016). Gut microbiota role in dietary protein metabolism and health-related outcomes: The two sides of the coin. Trends Food Sci. Technol..

[51] Whitt DD, Demoss RD (1975). Effect of microflora on the free amino acid distribution in various regions of the mouse gastrointestinal tract. Appl. Microbiol..

[52] Davila AM (2013). Re-print of "Intestinal luminal nitrogen metabolism: Role of the gut microbiota and consequences for the host". Pharmacol. Res..

[53] Diether NE, Willing BP (2019). Microbial fermentation of dietary protein: An important factor in diet–microbe–host interaction. Microorganisms.

[54] Gao J (2018). Impact of the gut microbiota on intestinal immunity mediated by tryptophan metabolism. Front. Cell. Infect. Microbiol..

[55] Mardinoglu A (2015). The gut microbiota modulates host amino acid and glutathione metabolism in mice. Mol. Syst. Biol..

[56] Dempsey J, Zhang A, Cui JY (2018). Coordinate regulation of long non-coding RNAs and protein-coding genes in germ-free mice. BMC Genom..

[57] Thwaites DT (2002). H+/dipeptide absorption across the human intestinal epithelium is controlled indirectly via a functional Na+/H+ exchanger. Gastroenterology.

[58] Bergen WG, Wu GJT (2009). Intestinal nitrogen recycling and utilization in health and disease. J. Nutr..

[59] de Vos WM, Tilg H, Van Hul M, Cani PD (2022). Gut microbiome and health: Mechanistic insights. Gut.

[60] Zhang P (2019). The distribution of tryptophan-dependent indole-3-acetic acid synthesis pathways in bacteria unraveled by large-scale genomic analysis. Molecules.

[61] Roager HM, Licht TR (2018). Microbial tryptophan catabolites in health and disease. Nat. Commun..

[62] Gutiérrez-Vázquez C, Quintana FJ (2018). Regulation of the immune response by the aryl hydrocarbon receptor. Immunity.

[63] Noerman S (2020). Associations of the serum metabolite profile with a healthy Nordic diet and risk of coronary artery disease. Clin. Nutr..

[64] Tuomainen M (2018). Associations of serum indolepropionic acid, a gut microbiota metabolite, with type 2 diabetes and low-grade inflammation in high-risk individuals. Nutr. Diabetes.

[65] de Mello VD (2017). Indolepropionic acid and novel lipid metabolites are associated with a lower risk of type 2 diabetes in the Finnish Diabetes Prevention Study. Sci. Rep..

[66] Yalçin A, Şarkici G, Kolaç UK (2020). PKR inhibitors suppress endoplasmic reticulum stress and subdue glucolipotoxicity-mediated impairment of insulin secretion in pancreatic beta cells. Turk. J. Biol..

[67] Riggio O (2010). Peripheral and splanchnic indole and oxindole levels in cirrhotic patients: A study on the pathophysiology of hepatic encephalopathy. Am. J. Gastroenterol..

[68] Ström K, Sjögren J, Broberg A, Schnürer J (2002). *Lactobacillus plantarum* MiLAB 393 produces the antifungal cyclic dipeptides cyclo (L-Phe-L-Pro) and cyclo (L-Phe-trans-4-OH-L-Pro) and 3-phenyllactic acid. Appl. Environ. Microbiol..

[69] Sivsammye G, Sims HV (1990). Presumptive identification of *Clostridium difficile* by detection of p-cresol in prepared peptone yeast glucose broth supplemented with p-hydroxyphenylacetic acid. J. Clin. Microbiol..

[70] Curtius HC, Mettler M, Ettlinger L (1976). Study of the intestinal tyrosine metabolism using stable isotopes and gas chromatography-mass spectrometry. J. Chromatogr..

[71] Saito Y, Sato T, Nomoto K, Tsuji H (2018). Identification of phenol- and p-cresol-producing intestinal bacteria by using media supplemented with tyrosine and its metabolites. FEMS Microbiol. Ecol..

[72] Ramakrishna BS (1989). Estimation of phenolic conjugation by colonic mucosa. J. Clin. Pathol..

[73] Schepers E, Glorieux G, Vanholder R (2010). The gut: The forgotten organ in uremia?. Blood Purif..

[74] Oliphant K, Allen-Vercoe E (2019). Macronutrient metabolism by the human gut microbiome: Major fermentation by-products and their impact on host health. Microbiome.

[75] Koistinen VM (2019). Contribution of gut microbiota to metabolism of dietary glycine betaine in mice and in vitro colonic fermentation. Microbiome.

[76] Xu W-Y (2019). 2-Aminoadipic acid protects against obesity and diabetes. J. Endocrinol..

[77] Wang TJ (2013). 2-Aminoadipic acid is a biomarker for diabetes risk. J. Clin. Investig..

[78] Matsumoto M (2012). Impact of intestinal microbiota on intestinal luminal metabolome. Sci. Rep..

[79] Nakamura A (2021). Symbiotic polyamine metabolism regulates epithelial proliferation and macrophage differentiation in the colon. Nat. Commun..

[80] Slezak K (2014). Association of germ-free mice with a simplified human intestinal microbiota results in a shortened intestine. Gut Microbes.

[81] Grover M, Kashyap PC (2014). Germ-free mice as a model to study effect of gut microbiota on host physiology. Neurogastroenterol. Motil..

[82] Tofalo R, Cocchi S, Suzzi G (2019). Polyamines and gut microbiota. Front. Nutr..

[83] Koppe L, Fouque D, Soulage CO (2018). The role of gut microbiota and diet on uremic retention solutes production in the context of chronic kidney disease. Toxins.

[84] Saheki T (1980). Comparison of the urea cycle in conventional and germ-free mice. J. Biochem..

[85] Qi H (2019). Lactobacillus maintains healthy gut mucosa by producing L-Ornithine. Commun. Biol..

[86] Vissers S, Legrain C, Wiame JM (1986). Control of a futile urea cycle by arginine feedback inhibition of ornithine carbamoyltransferase in *Agrobacterium tumefaciens* and Rhizobia. Eur. J. Biochem..

[87] Hobley L (2014). Norspermidine is not a self-produced trigger for biofilm disassembly. Cell.

[88] Staley C, Weingarden AR, Khoruts A, Sadowsky MJ (2017). Interaction of gut microbiota with bile acid metabolism and its influence on disease states. Appl. Microbiol. Biotechnol..

[89] Baier V (2019). A physiology-based model of human bile acid metabolism for predicting bile acid tissue levels after drug administration in healthy subjects and BRIC type 2 patients. Front. Physiol..

[90] Mistry RH, Verkade HJ, Tietge UJ (2017). Reverse cholesterol transport is increased in germ-free mice-brief report. Arterioscler. Thromb. Vasc. Biol..

[91] Kaur H, Seeger D, Golovko S, Golovko M, Combs CK (2021). Liver bile acid changes in mouse models of Alzheimer’s disease. Int. J. Mol. Sci..

[92] Ay Ü (2022). New kids on the block: Bile salt conjugates of microbial origin. Metabolites.

[93] Gonzalez FJ, Jiang C, Bisson WH, Patterson AD (2015). Inhibition of farnesoid X receptor signaling shows beneficial effects in human obesity. J. Hepatol..

[94] Gonzalez FJ, Jiang C, Patterson AD (2016). An intestinal microbiota-farnesoid X receptor axis modulates metabolic disease. Gastroenterology.

[95] Smith SA (2021). Mitochondrial dysfunction in inflammatory bowel disease alters intestinal epithelial metabolism of hepatic acylcarnitines. J. Clin. Investing..

[96] Schooneman MG, Vaz FM, Houten SM, Soeters MR (2013). Acylcarnitines: Reflecting or inflicting insulin resistance?. Diabetes.

[97] Yan ZX (2018). Fecal microbiota transplantation in experimental ulcerative colitis reveals associated gut microbial and host metabolic reprogramming. Appl. Environ. Microbiol..

[98] Wu W-K (2019). Identification of TMAO-producer phenotype and host–diet–gut dysbiosis by carnitine challenge test in human and germ-free mice. Gut.

[99] Rajakovich LJ, Fu B, Bollenbach M, Balskus EP (2021). Elucidation of an anaerobic pathway for metabolism of l-carnitine–derived & #x3b3;-butyrobetaine to trimethylamine in human gut bacteria. Proc. Natl. Acad. Sci..

[100] Al-Waiz M, Mikov M, Mitchell SC, Smith RL (1992). The exogenous origin of trimethylamine in the mouse. Metab. Clin. Exp..

[101] Bremer J (1983). Carnitine–metabolism and functions. Physiol. Rev..

[102] Hussain MB (2019). Plant Physiological Aspects of Phenolic Compounds.

[103] Crespillo A (2011). Reduction of body weight, liver steatosis and expression of stearoyl-CoA desaturase 1 by the isoflavone daidzein in diet-induced obesity. Br. J. Pharmacol..

[104] Gaya P, Peirotén Á, Landete JM (2017). Transformation of plant isoflavones into bioactive isoflavones by lactic acid bacteria and bifidobacteria. J. Funct. Foods.

[105] Rawat S (2019). Recent updates on daidzein against oxidative stress and cancer. EXCLI J..

[106] Hasyima Omar M, González Barrio R, Pereira-Caro G, Almutairi TM, Crozier A (2020). In vitro catabolism of 3′,4′-dihydroxycinnamic acid by human colonic microbiota. Int. J. Food Sci. Nutr..

[107] Chen JR (2019). 3-(3-Hydroxyphenyl)-propionic acid (PPA) suppresses osteoblastic cell senescence to promote bone accretion in mice. JBMR Plus.

[108] Rowland I (2018). Gut microbiota functions: Metabolism of nutrients and other food components. Eur. J. Nutr..

[109] Santana-Gálvez J, Villela Castrejón J, Serna-Saldívar SO, Jacobo-Velázquez DA (2020). Anticancer potential of dihydrocaffeic acid: A chlorogenic acid metabolite. CyTA J. Food.

[110] Pekkinen J (2014). Disintegration of wheat aleurone structure has an impact on the bioavailability of phenolic compounds and other phytochemicals as evidenced by altered urinary metabolite profile of diet-induced obese mice. Nutr. Metab. (Lond).

[111] Nguyen TLA, Vieira-Silva S, Liston A, Raes J (2015). How informative is the mouse for human gut microbiota research?. Dis. Model. Mech..

[112] Uzbay T (2019). Germ-free animal experiments in the gut microbiota studies. Curr. Opin. Pharmacol..

[113] Pekkinen J (2014). Disintegration of wheat aleurone structure has an impact on the bioavailability of phenolic compounds and other phytochemicals as evidenced by altered urinary metabolite profile of diet-induced obese mice. Nutr. Metab..

[114] Hanhineva K (2014). The postprandial plasma rye fingerprint includes benzoxazinoid-derived phenylacetamide sulfates. J. Nutr..

[115] Pekkinen J (2013). Betaine supplementation causes increase in carnitine metabolites in the muscle and liver of mice fed a high-fat diet as studied by nontargeted LC-MS metabolomics approach. Mol. Nutr. Food Res..

[116] Tsugawa H (2015). MS-DIAL: Data-independent MS/MS deconvolution for comprehensive metabolome analysis. Nat. Methods.

[117] Pang Z, Chong J, Li S, Xia J (2020). MetaboAnalystR 3.0: Toward an optimized workflow for global metabolomics. Metabolites.

