# Supplementary material for: Tissue-wide metabolomics reveals wide impact of gut microbiota on mice metabolite composition
Source: Sci Rep. 2022 Sep 2;12:15018. doi: 10.1038/s41598-022-19327-w (PMC9440220; doi:10.1038/s41598-022-19327-w)
Supplement: Supplementary file 1 — Supplementary Information 1. [file 41598_2022_19327_MOESM1_ESM.pptx]

## Slide 1
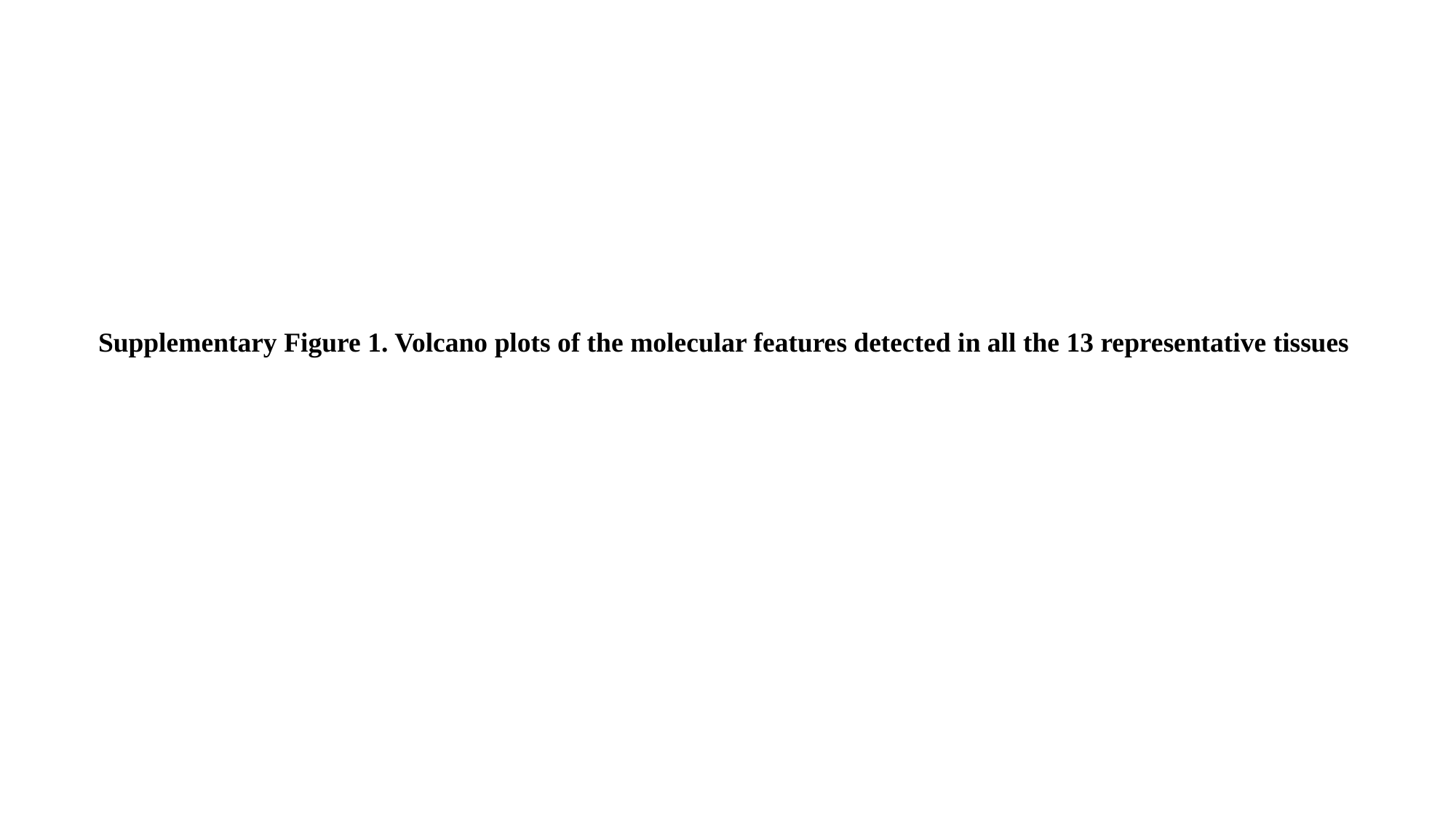

Supplementary Figure 1. Volcano plots of the molecular features detected in all the 13 representative tissues

## Slide 2
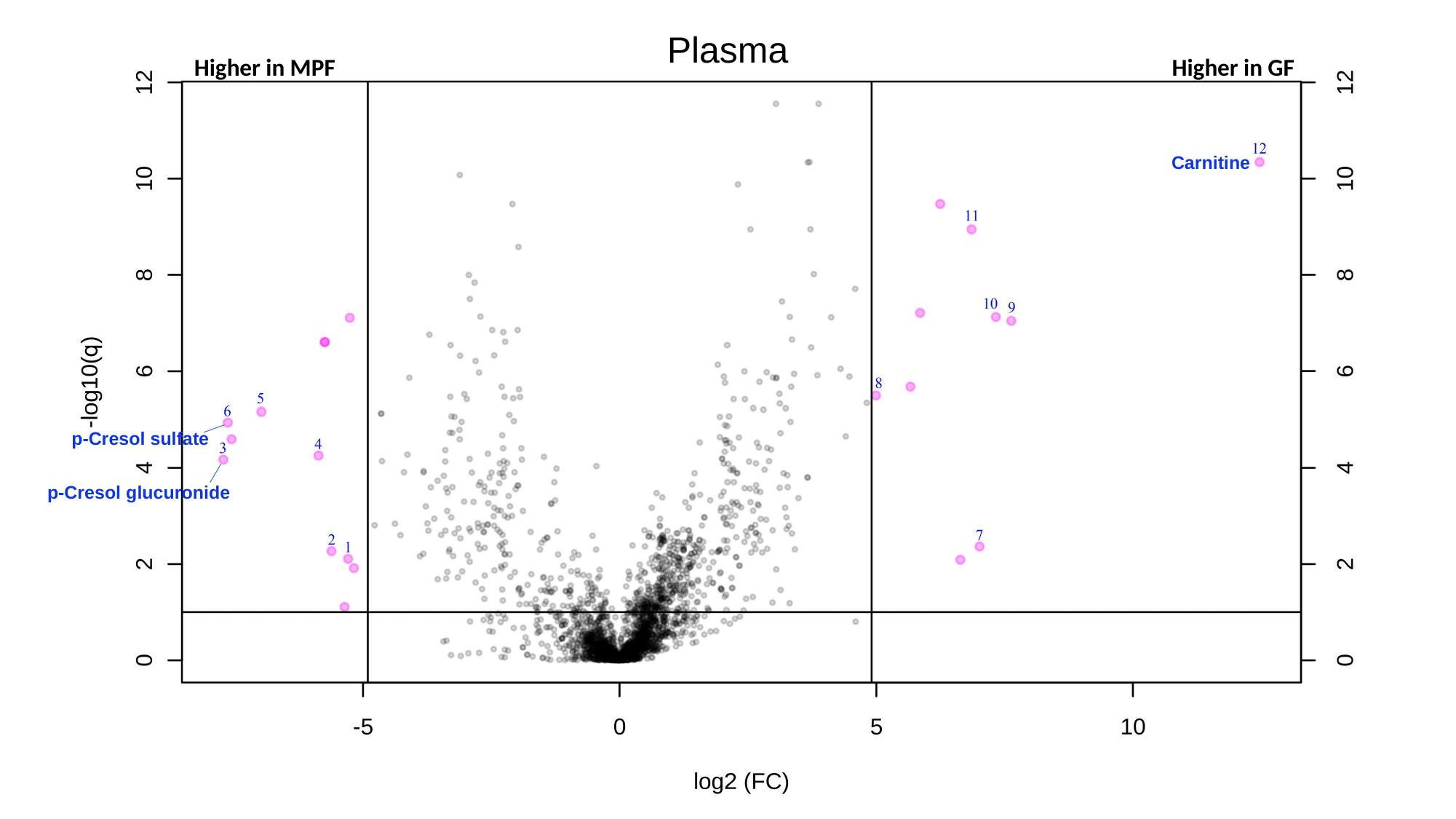

Plasma
Carnitine
p-Cresol sulfate
p-Cresol glucuronide
Higher in MPF
Higher in GF

## Slide 3
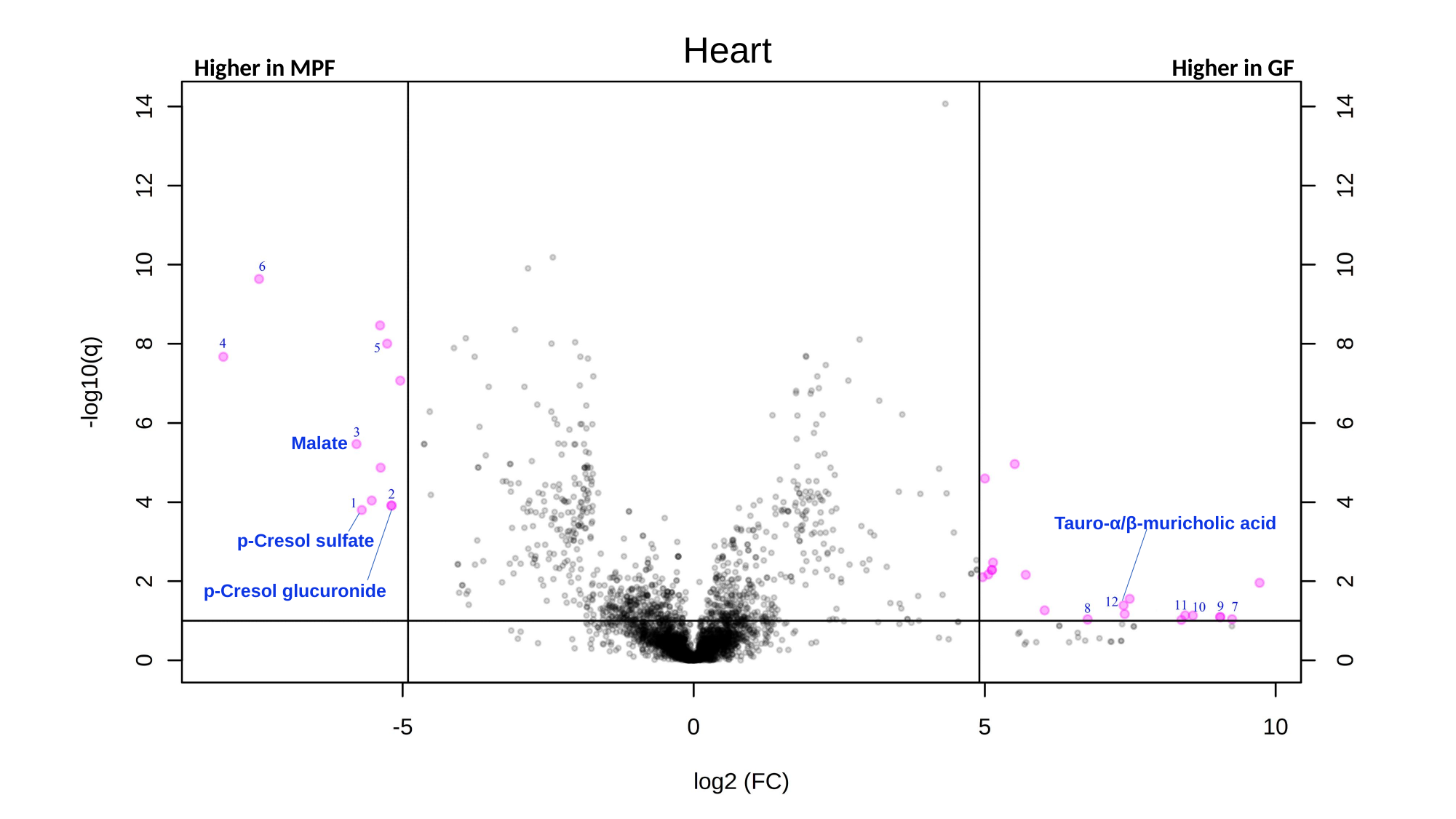

Heart
p-Cresol sulfate
p-Cresol glucuronide
Higher in MPF
Higher in GF
Malate
Tauro-α/β-muricholic acid

## Slide 4
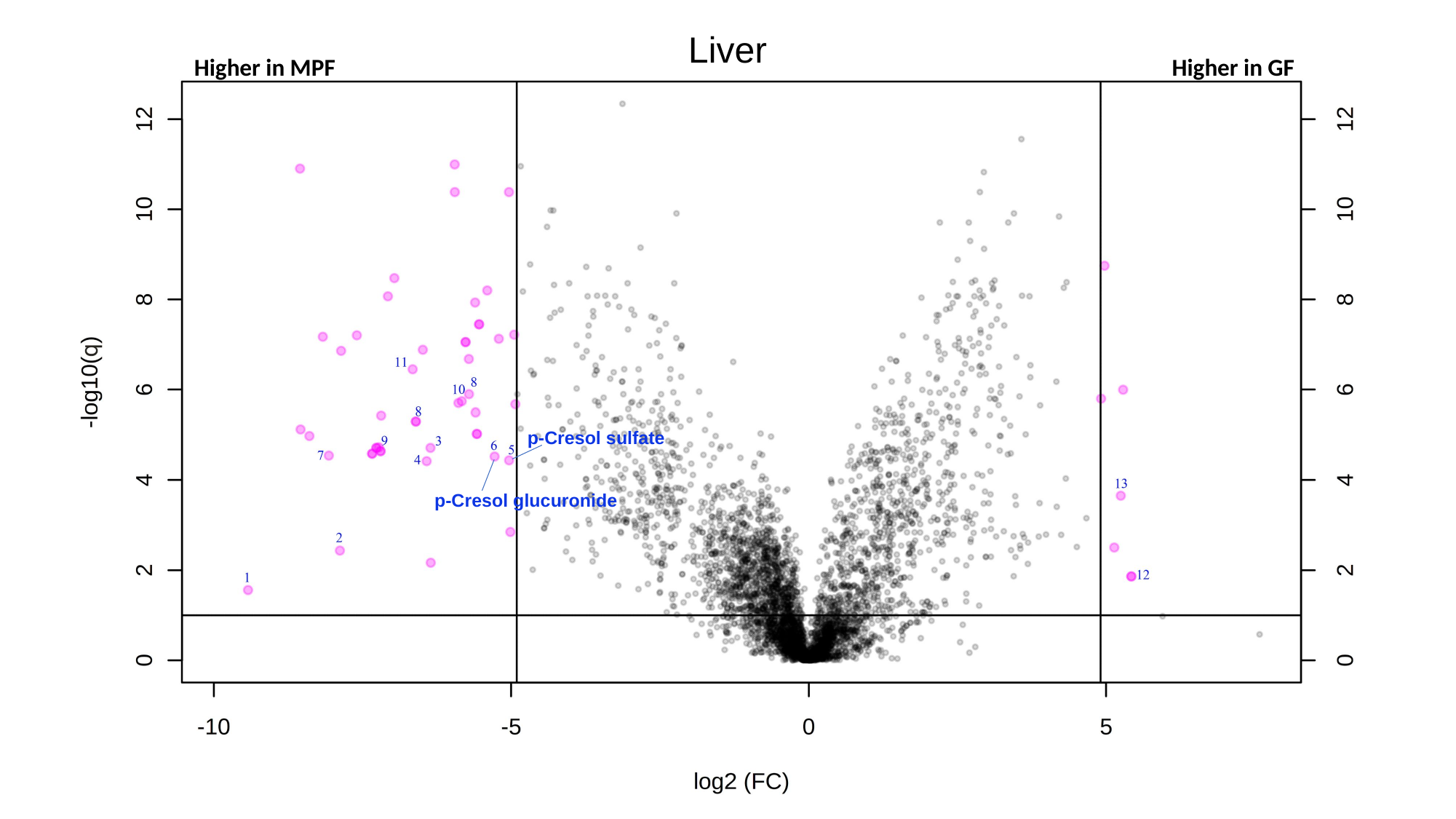

Liver
p-Cresol sulfate
p-Cresol glucuronide
Higher in MPF
Higher in GF

## Slide 5
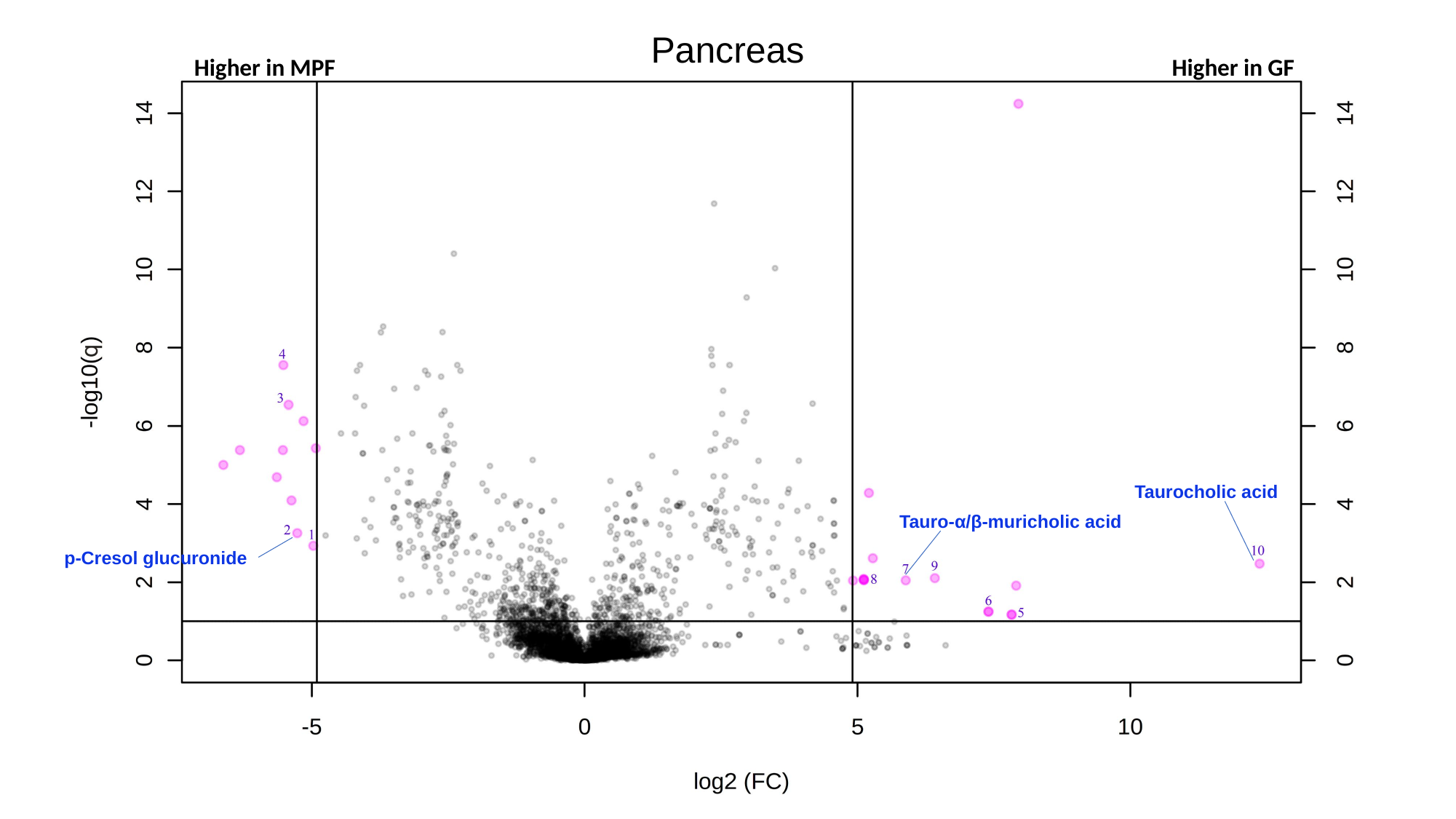

Pancreas
Taurocholic acid
p-Cresol glucuronide
Higher in MPF
Higher in GF
Tauro-α/β-muricholic acid

## Slide 6
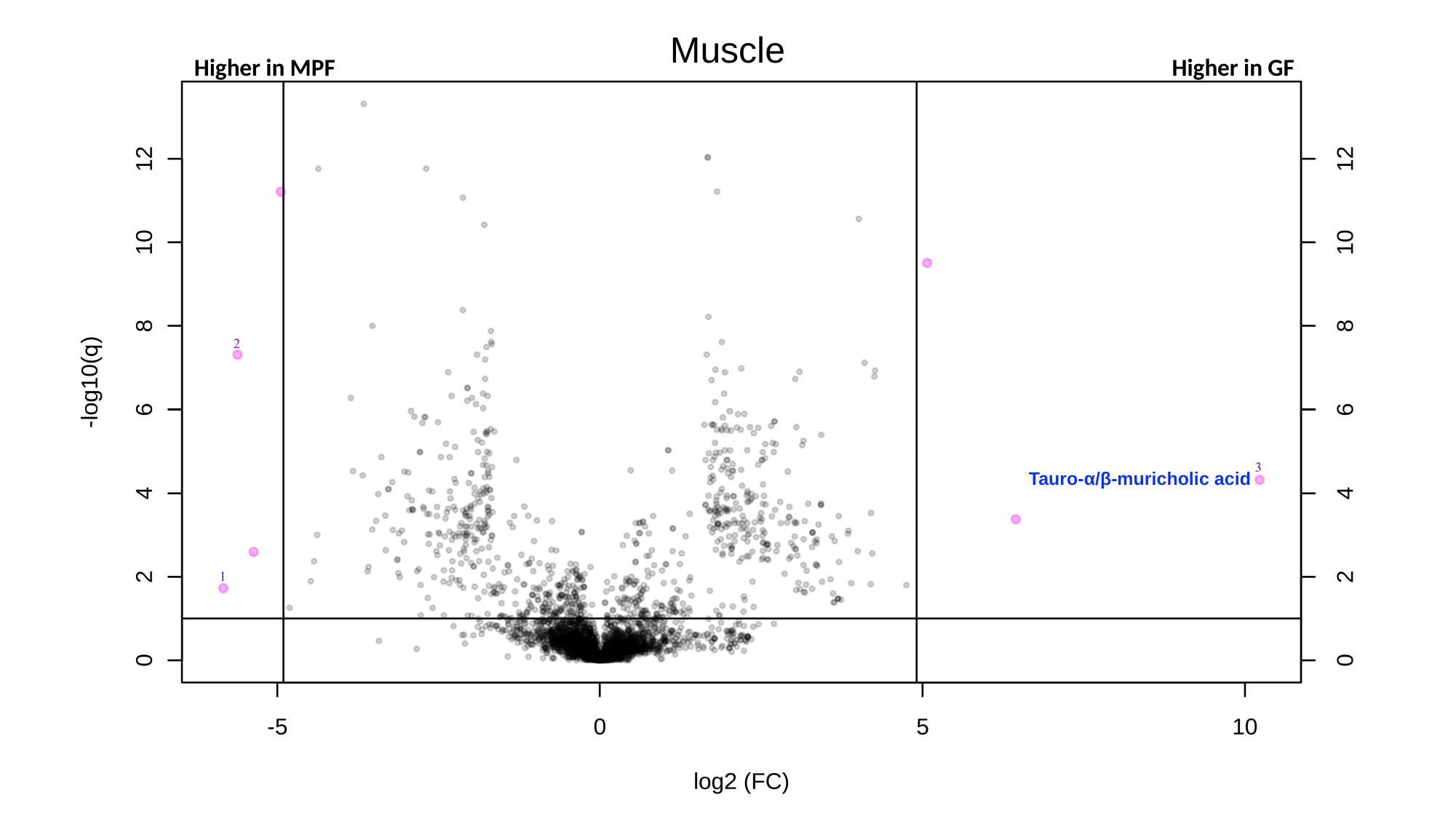

Muscle
Tauro-α/β-muricholic acid
Higher in MPF
Higher in GF

## Slide 7
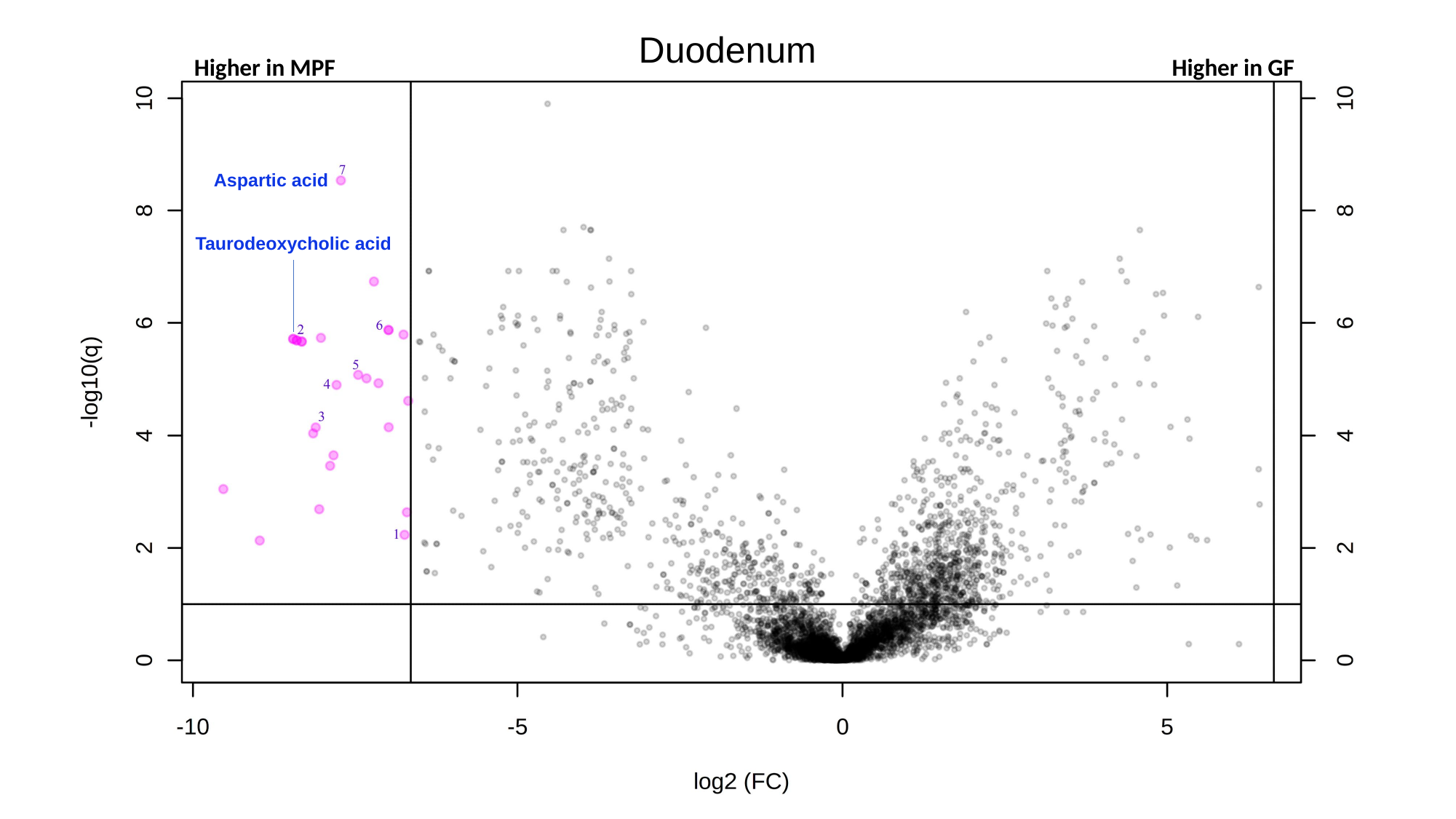

Duodenum
Higher in MPF
Higher in GF
Aspartic acid
Taurodeoxycholic acid

## Slide 8
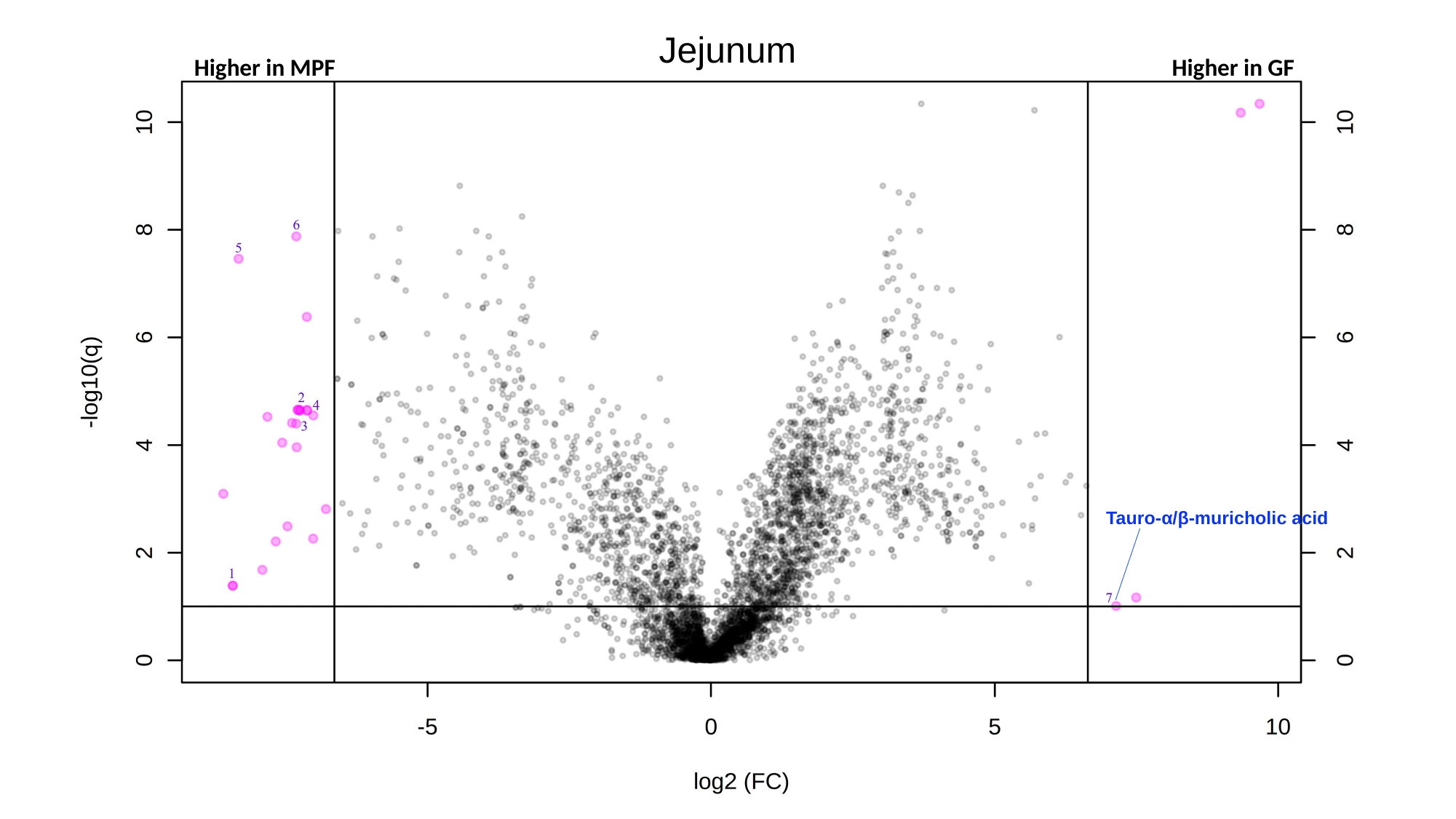

Jejunum
Higher in MPF
Higher in GF
Tauro-α/β-muricholic acid

## Slide 9
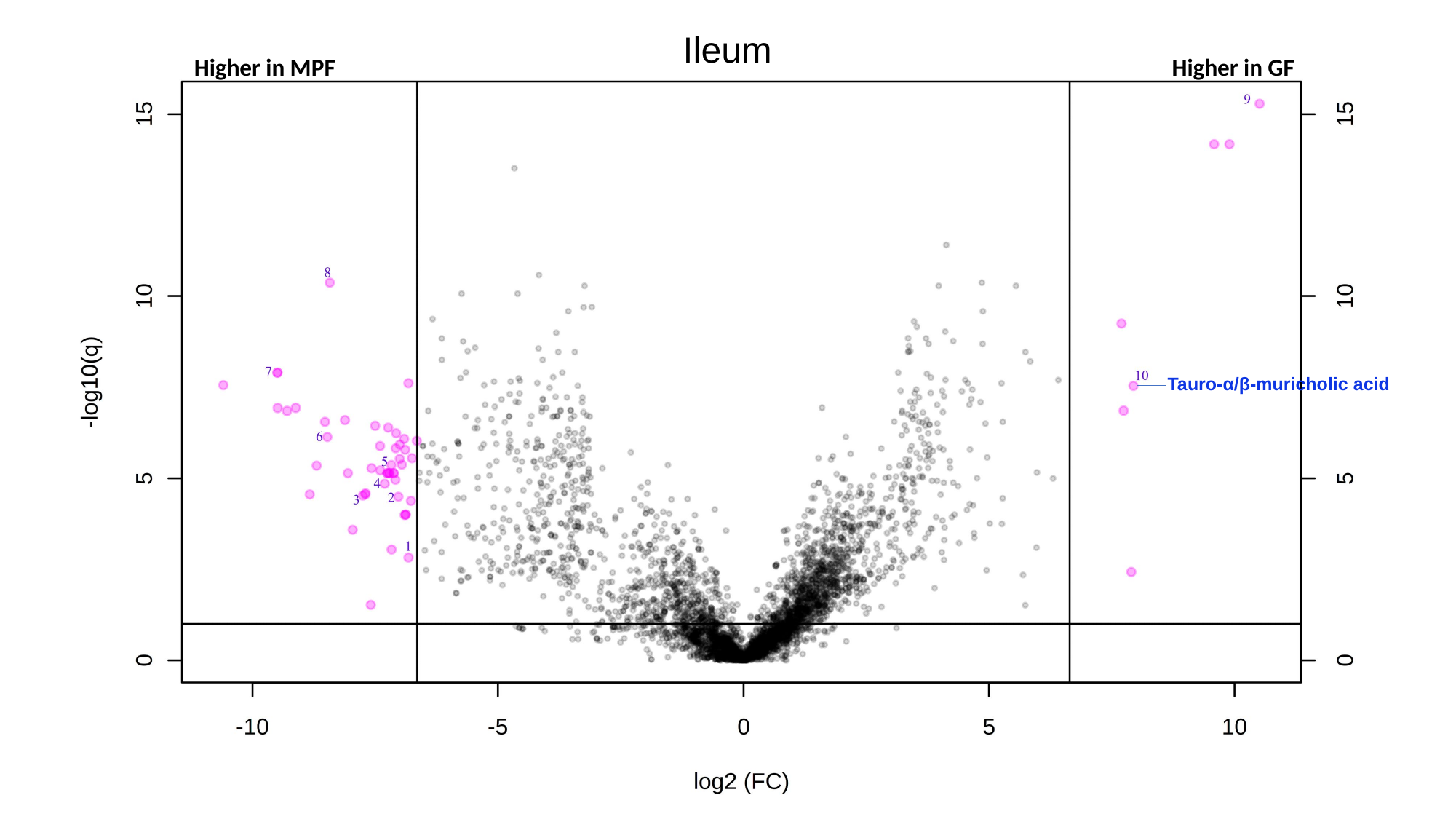

Ileum
Higher in MPF
Higher in GF
Tauro-α/β-muricholic acid

## Slide 10
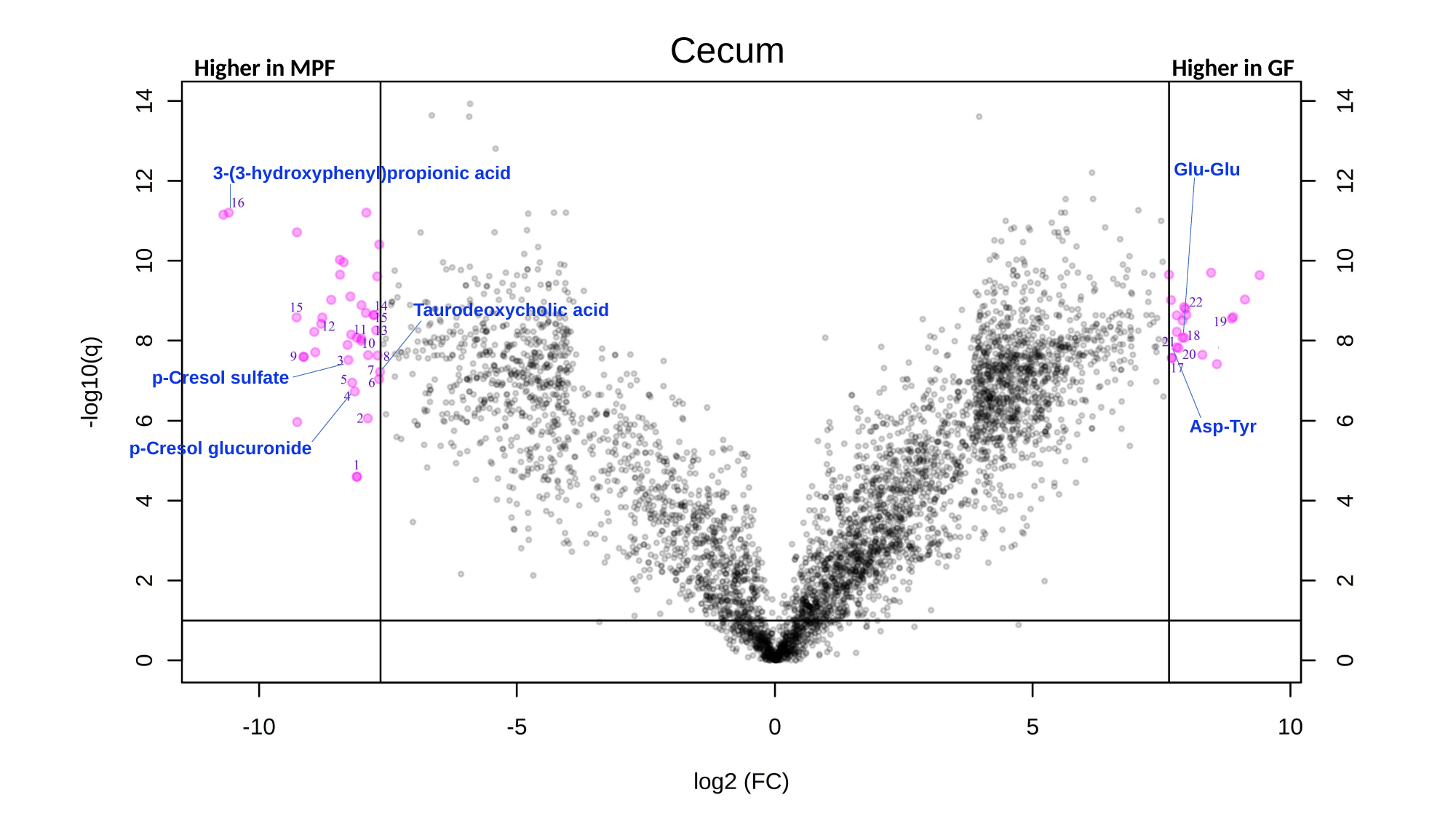

Cecum
Taurodeoxycholic acid
p-Cresol sulfate
p-Cresol glucuronide
Higher in MPF
Higher in GF
3-(3-hydroxyphenyl)propionic acid
Glu-Glu
Asp-Tyr

## Slide 11
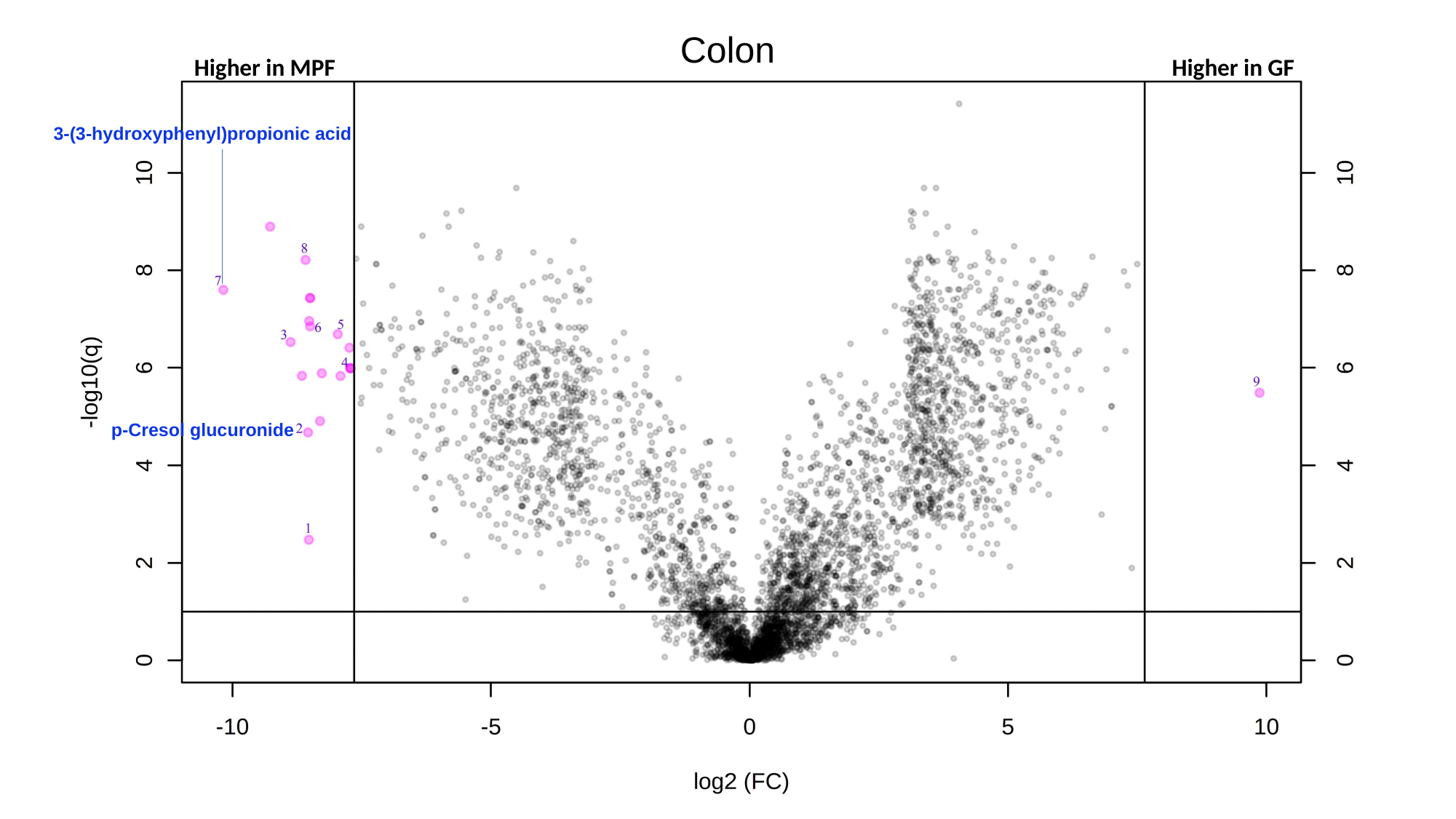

Colon
Higher in MPF
Higher in GF
3-(3-hydroxyphenyl)propionic acid
p-Cresol glucuronide

## Slide 12
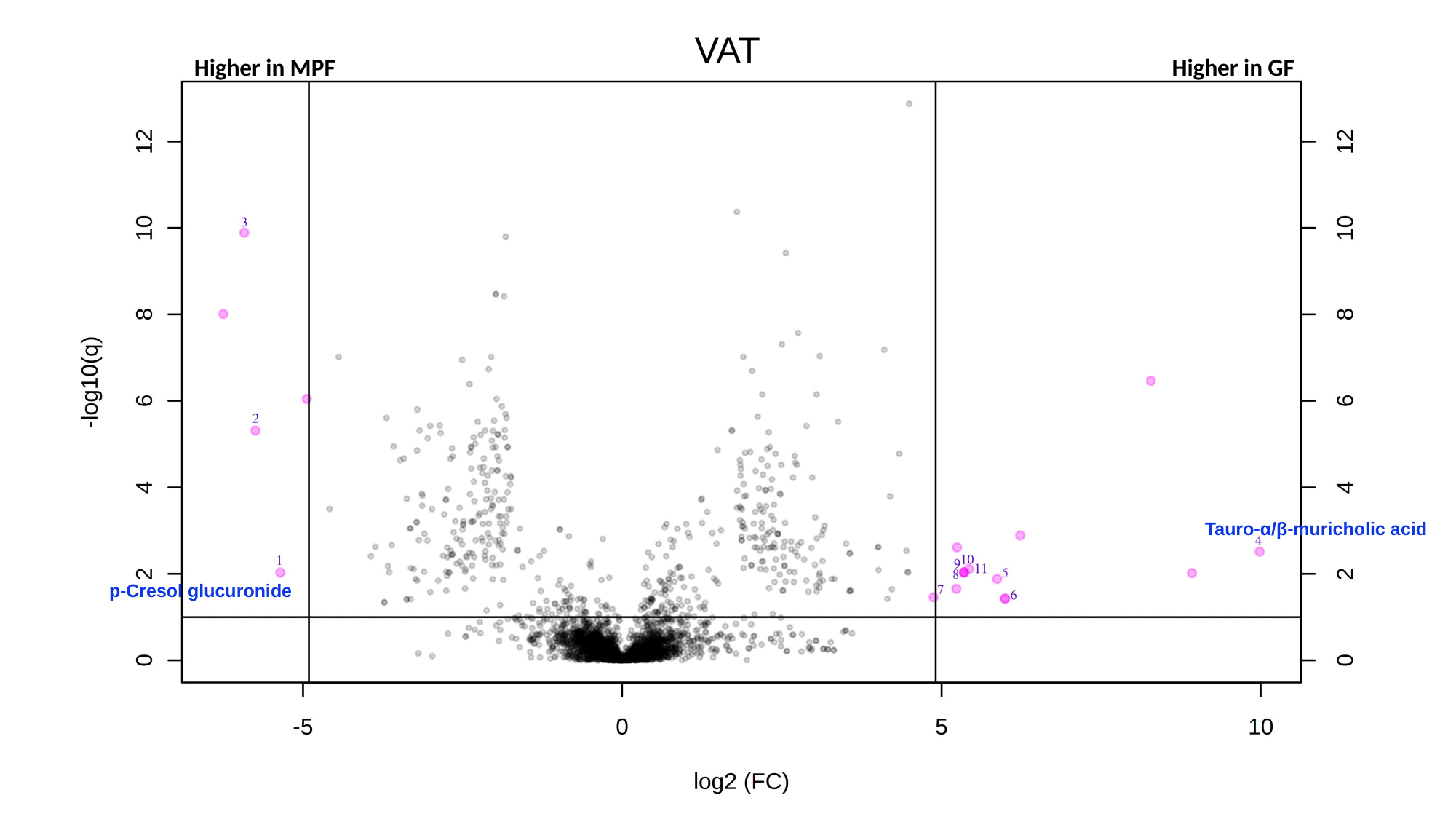

VAT
Higher in MPF
Higher in GF
Tauro-α/β-muricholic acid
p-Cresol glucuronide

## Slide 13
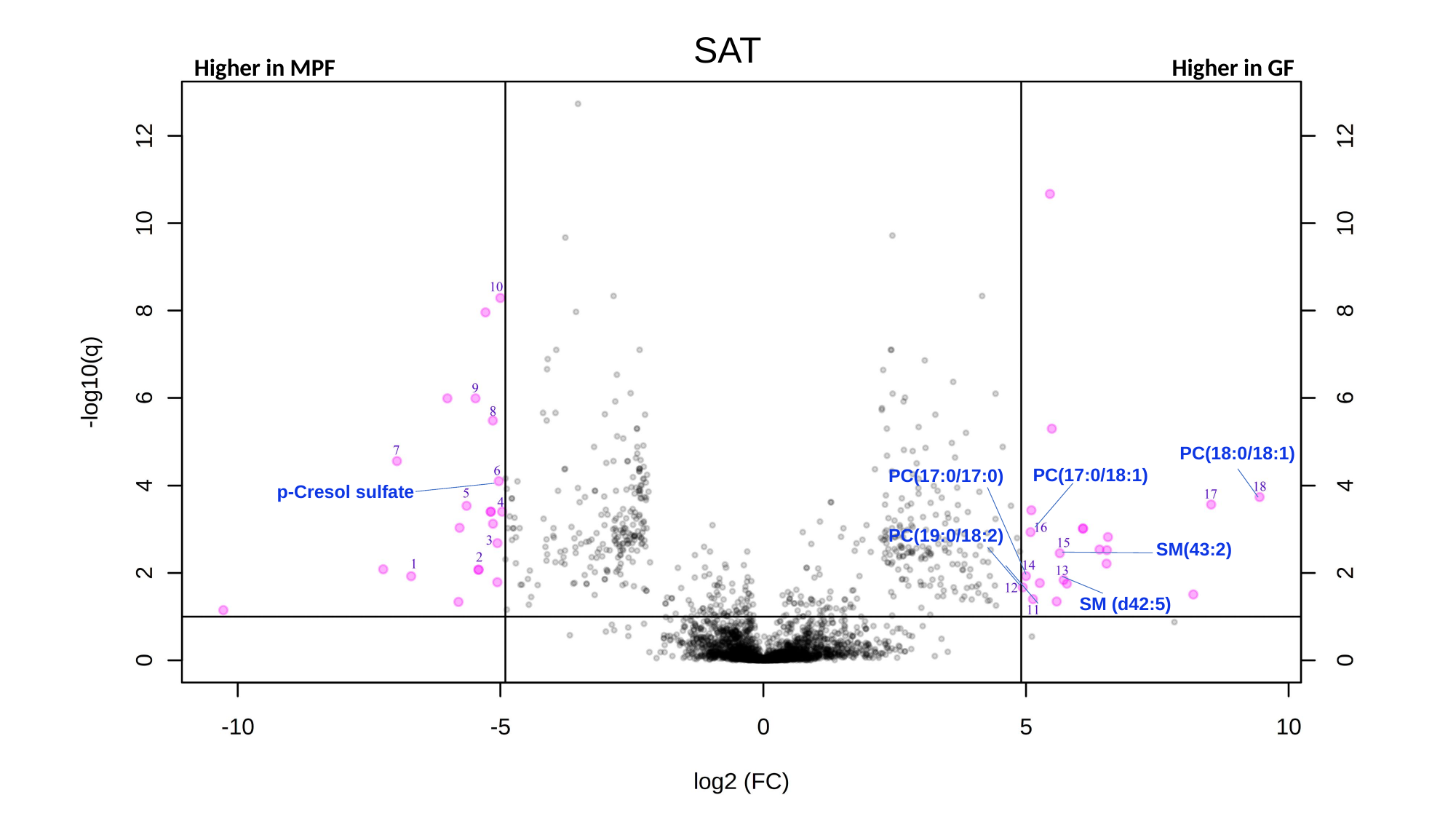

SAT
p-Cresol sulfate
Higher in MPF
Higher in GF
PC(18:0/18:1)
PC(17:0/18:1)
PC(17:0/17:0)
PC(19:0/18:2)
SM(43:2)
SM (d42:5)

## Slide 14
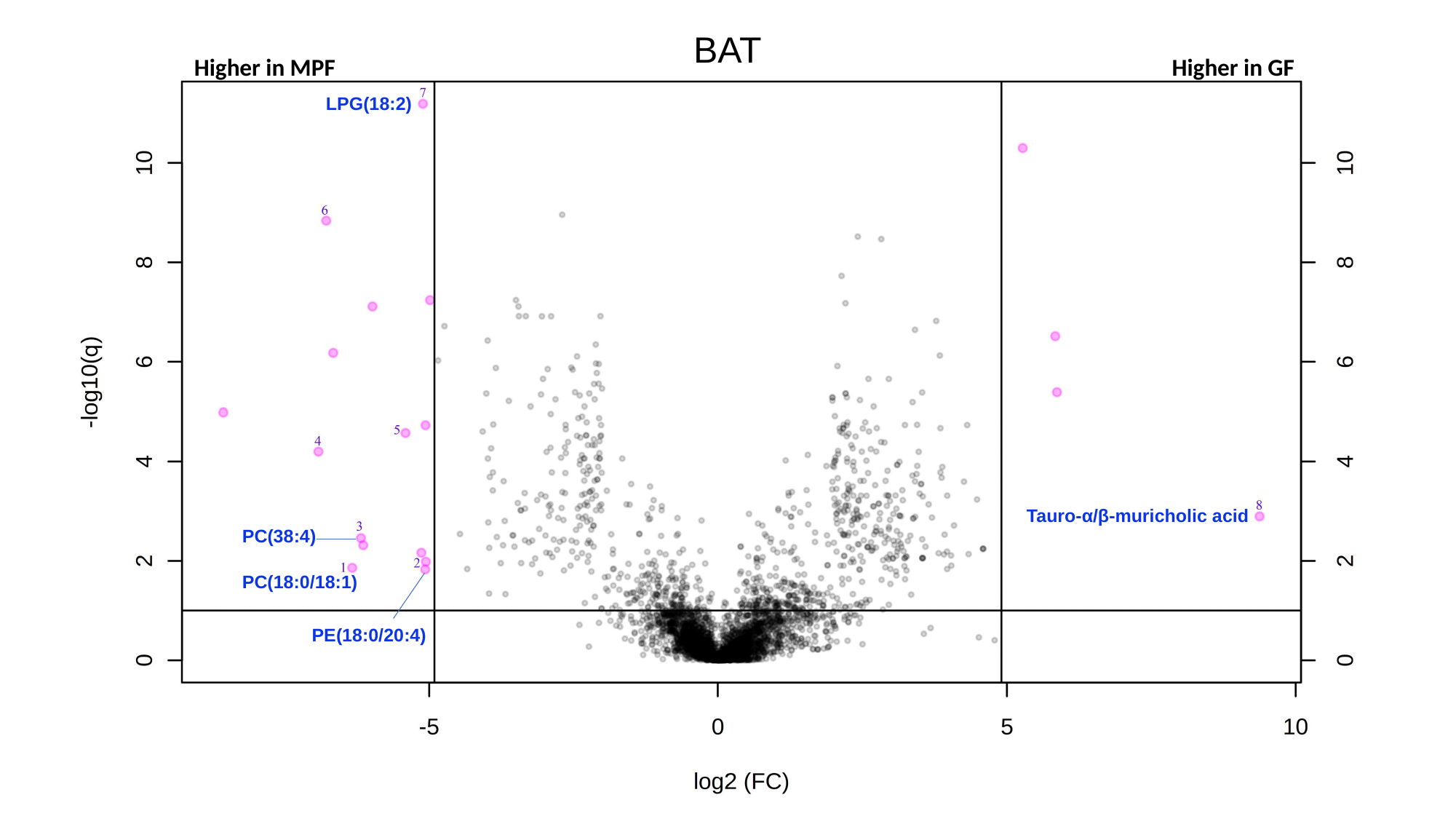

BAT
Tauro-α/β-muricholic acid
Higher in MPF
Higher in GF
LPG(18:2)
PC(38:4)
PC(18:0/18:1)
PE(18:0/20:4)
