# Supplementary material for: Tissue-wide metabolomics reveals wide impact of gut microbiota on mice metabolite composition
Source: Sci Rep. 2022 Sep 2;12:15018. doi: 10.1038/s41598-022-19327-w (PMC9440220; doi:10.1038/s41598-022-19327-w)
Supplement: Supplementary file 2 — Supplementary Information 2. [file 41598_2022_19327_MOESM2_ESM.docx]

**Supplementary Figure 2. *k*-means cluster analysis of the filtered* molecular features (4,605 molecular features).** Clusters 2, 4, and 5 containing 1,214 molecular features were considered for compound identification. Clusters 2 and 4 showed the molecular features that were mostly differential in the cecum and the colon and were higher in abundance in the GF and MPF mice, respectively. Cluster 5 showed the molecular features that were mostly differential in the duodenum, jejunum, ileum, and liver and were higher in abundance in the MPF mice. *Filtering criteria for inclusion were (1) p-value ≤0.05, and q-value ≤ 0.05, (2) high-intensity metabolite values (raw abundance ≥ 100,000), (3) containment of MS/MS fragmentation, (4) and retention time ≥0.7 min. For a molecular feature to be included, these inclusion criteria should exist at least in one tissue and one mouse group (GF or MPF). The different mouse groups are illustrated as follows with 5 mice in each group: Plasma MPF, Plasma GF, Heart MPF, Heart GF, Liver MPF, Liver GF, Pancreas MPF, Pancreas GF, Muscle MPF, Muscle GF, Duodenum MPF, Duodenum GF, Jejunum MPF, Jejunum GF, Ileum MPF, Ileum GF, Cecum MPF, Cecum GF, Colon MPF, Colon GF, VAT MPF, VAT GF, SAT MPF, SAT GF, BAT MPF, and BAT GF.
