# Supplementary material for: Tissue-wide metabolomics reveals wide impact of gut microbiota on mice metabolite composition
Source: Sci Rep. 2022 Sep 2;12:15018. doi: 10.1038/s41598-022-19327-w (PMC9440220; doi:10.1038/s41598-022-19327-w)
Supplement: Supplementary file 3 — Supplementary Information 3. [file 41598_2022_19327_MOESM3_ESM.docx]

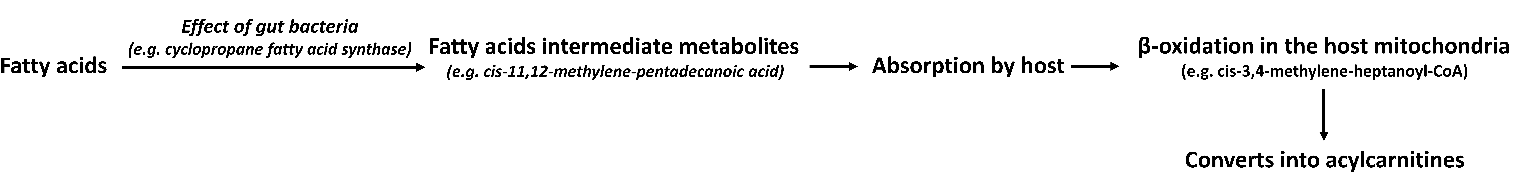


**Supplementary Figure 3.** Induction of acylcarnitine production in the MPF mice (a suggested mechanism).
