# Supplementary material for: Tissue-wide metabolomics reveals wide impact of gut microbiota on mice metabolite composition
Source: Sci Rep. 2022 Sep 2;12:15018. doi: 10.1038/s41598-022-19327-w (PMC9440220; doi:10.1038/s41598-022-19327-w)
Supplement: Supplementary file 4 — Supplementary Information 4. [file 41598_2022_19327_MOESM4_ESM.docx]

| **Supplementary Table 1. Summary of detected molecular features in all tissues from all the ionization modes** | | | | | | | | | | | | | | | | |
| --- | --- | --- | --- | --- | --- | --- | --- | --- | --- | --- | --- | --- | --- | --- | --- | --- |
| **Tissue** | **Total detected molecular features^#^** | | **Total significantly- changed molecular features*** | | **Significant change MPF > GF^†^** | | **Significant change GF > MPF^†^** | | **Significant unique to MPF^†^** | | **Significant unique to GF^†^** | | **Significant Shared^†^** | | |  |
| **Cecum** | 17894 | (74%) | 11375 | (64%) | 4603 | (40%) | 6772 | (60%) | 2438 | (21%) | 3621 | (32%) | 5315 | (47%) |  |  |
| **Ileum** | 16659 | (69%) | 6074 | (36%) | 2198 | (36%) | 3876 | (64%) | 1419 | (23%) | 2271 | (38%) | 2380 | (39%) |  |  |
| **Duodenum** | 16046 | (66%) | 4149 | (26%) | 1713 | (41%) | 2436 | (59%) | 1299 | (31%) | 1505 | (36%) | 1342 | (33%) |  |  |
| **Jejunum** | 15613 | (64%) | 6247 | (40%) | 2257 | (36%) | 3990 | (64%) | 1321 | (21%) | 2489 | (40%) | 2500 | (40%) |  |  |
| **Colon** | 15004 | (62%) | 7226 | (48%) | 2448 | (34%) | 4778 | (66%) | 1737 | (24%) | 3606 | (50%) | 1880 | (26%) |  |  |
| **Liver** | 10021 | (41%) | 3715 | (37%) | 2295 | (62%) | 1399 | (38%) | 1055 | (28%) | 628 | (17%) | 2032 | (55%) |  |  |
| **Pancreas** | 9415 | (39%) | 1625 | (17%) | 982 | (60%) | 643 | (40%) | 672 | (41%) | 440 | (27%) | 513 | (32%) |  |  |
| **SAT** | 8976 | (37%) | 1373 | (15%) | 814 | (61%) | 525 | (39%) | 756 | (56%) | 471 | (35%) | 112 | (9%) |  |  |
| **BAT** | 8687 | (36%) | 1409 | (16%) | 665 | (47%) | 744 | (53%) | 494 | (35%) | 613 | (44%) | 302 | (21%) |  |  |
| **Heart** | 7748 | (32%) | 1233 | (16%) | 646 | (52%) | 587 | (48%) | 434 | (35%) | 397 | (32%) | 402 | (33%) |  |  |
| **Muscle** | 7145 | (29%) | 1257 | (18%) | 675 | (54%) | 582 | (46%) | 524 | (42%) | 475 | (38%) | 258 | (21%) |  |  |
| **VAT** | 7019 | (29%) | 981 | (14%) | 525 | (54%) | 456 | (46%) | 434 | (44%) | 356 | (36%) | 191 | (20%) |  |  |
| **Plasma** | 5469 | (23%) | 1061 | (19%) | 402 | (38%) | 659 | (62%) | 330 | (31%) | 397 | (37%) | 334 | (32%) |  |  |
| **VAT** (visceral adipose tissue), **SAT** (subcutaneous adipose tissue), **BAT** (brown adipose tissue)  ^#^The percentage is based on the total number of molecular features detected in all the tissues from the ionization modes (i.e., 24,294).  *Defined as having a fold-change ≥ 1.3, *p*-value ≤0.05, and *q*-value ≤ 0.05.  **^†^**The percentage is based on the tissue-specific number of the total significantly changed molecular features | | | | | | | | | | | | | | | | |
